# A LNK–CBL–HNRPA2B1–GPX4 signaling axis mediates dopaminergic neuron vulnerability to ferroptosis in Parkinson's disease

**DOI:** 10.1016/j.redox.2026.104039

**Published:** 2026-01-23

**Authors:** Ziqi Liu, Ruoxun Wang, Min Shen, Xinrui Lan, Weixing Yan, Sainan Wang, Mingfeng Jiang, Rongqing Li, Jie Zhao, Qicheng Wang, Xinyi Xv, Jingwen Zhou, Xin Pan, Wei Li, Weijuan Gong, Li Qian

**Affiliations:** a Key Laboratory of the Jiangsu Higher Education Institutions for Nucleic Acid & Cell Fate Regulation (Yangzhou University), Faculty of Medicine, Yangzhou University, Yangzhou, Jiangsu, 225001, China; b Jiangsu Key Laboratory of Immunity and Metabolism, Department of Pathogenic Biology and Immunology, Xuzhou Medical University, Xuzhou, Jiangsu, 221004, China; c Department of Cardiology, Institute of Cardiovascular Disease, Yangzhou Key Lab of Innovation Frontiers in Cardiovascular Disease, Affiliated Hospital of Yangzhou University, Yangzhou University, Yangzhou, Jiangsu, 225001, China; d Kunshan Hospital of Chinese Medicine, Affiliated Hospital of Yangzhou University, Kunshan, Jiangsu, 215300, China

**Keywords:** Parkinson's disease, LNK, Ferroptosis, Dopaminergic neurodegeneration, Lifitegrast

## Abstract

The upstream mechanisms governing neuronal susceptibility to ferroptosis in Parkinson's disease (PD) remain incompletely defined. This study investigates the molecular pathways mediating dopaminergic neuron vulnerability to ferroptosis in PD. The Lymphocyte adaptor protein (LNK) is identified as an upstream regulator, with its expression being significantly increased in peripheral blood of PD patients and positively associating with motor impairment severity. Similar upregulation occurs in murine PD models, coinciding with enhanced neuronal susceptibility. LNK interacts with the E3 ubiquitin ligase casitas B-lineage lymphoma proto-oncogene (CBL), promoting nuclear translocation and K27-linked polyubiquitination-driven degradation of the RNA-binding protein heterogeneous nuclear ribonucleoprotein A2/B1 (HNRNPA2B1). As an N6-methyladenosine (m6A) reader, HNRNPA2B1 stabilizes GPX4 transcripts, and its depletion reduces GPX4 levels, impairing glutathione-dependent lipid peroxidation defense. A pharmacological screen identifies lifitegrast an FDA-approved ophthalmic LFA-1 antagonist, as a putative small molecule modulator capable of interacting with the LNK SH2 domain and attenuating LNK-associated signaling in cellular assays. In PD models, lifitegrast administration or genetic ablation of LNK was observed to mitigate dopaminergic neurodegeneration. These findings define the LNK–CBL–HNRNPA2B1–GPX4 axis in ferroptotic regulation and support LNK as a potential therapeutic target in PD.

## Highlights

• LNK expression is elevated in Parkinson's disease and correlates with motor severity.
• Novel LNK–CBL–HNRNPA2B1–GPX4 axis regulates neuronal ferroptosis vulnerability.
• LNK promotes HNRNPA2B1 degradation, reducing GPX4 stability and enhancing ferroptosis.
• FDA-approved lifitegrast inhibits LNK and provides neuroprotection in PD models.

## Graphical abstract

**Figure:**
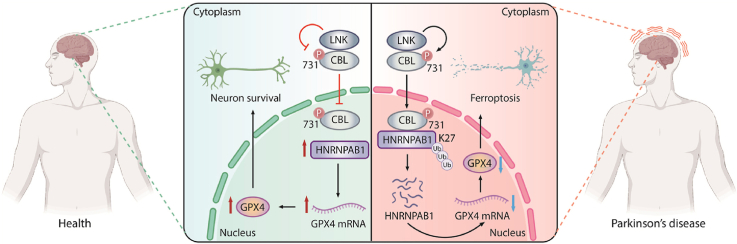

## 1 Introduction

Parkinson's disease (PD) is recognized as the second most common neurodegenerative disorder worldwide, affecting approximately 1 % of individuals older than 60 years [1]. The pathological hallmark of PD is the progressive degeneration of dopaminergic (DA) neurons in the substantia nigra pars compacta (SNpc), leading to motor dysfunction and, ultimately, severe disability [[2], [3], [4]]. Despite decades of investigation, interventions capable of altering disease course remain unavailable [5].

Emerging evidence indicates that ferroptosis—an iron-dependent, regulated form of cell death characterized by lipid peroxidation—is a critical mechanism underlying DA neuronal vulnerability in PD [[6], [7], [8]]. DA neurons are intrinsically susceptible to ferroptotic stress due to their high iron content, required for dopamine synthesis, and the inherently pro-oxidant nature of dopamine metabolism. Postmortem analyses of PD brains have demonstrated pronounced iron accumulation in the SNpc, accompanied by elevated lipid peroxidation markers, underscoring the clinical relevance of ferroptotic injury in PD [[9], [10], [11], [12]]. Central to ferroptosis regulation is glutathione peroxidase 4 (GPX4), a selenoenzyme that catalyzes the reduction of lipid hydroperoxides to their corresponding alcohols, thereby preventing the propagation of lipid peroxidation cascades [13,14]. In Parkinson's disease (PD), pathological hallmarks such as α-synuclein aggregation and neuroinflammation compromise GPX4 function by driving glutathione depletion, direct protein inactivation, and aberrant post-translational modifications [15,16]. These converging insults collectively compromise GPX4 activity, establishing it as a central nexus in ferroptotic regulation.

The lymphocyte adaptor protein (LNK), encoded by *SH2B3*, is a negative regulator of cytokine signaling critical for immune homeostasis. Its conserved SH2 domain directly binds phosphorylated Janus kinases (JAKs), thereby attenuating their activity and suppressing downstream STAT signaling [17,18]. Beyond the hematopoietic system, emerging evidence indicates that LNK also exerts regulatory effects within the central nervous system. Specifically, LNK serves as a negative modulator of nerve growth factor signaling by constraining TrkA receptor-mediated signaling cascades, consequently limiting neurite outgrowth in the rat pheochromocytoma cell line PC12 and cortical neurons [19].

Following ischemic stroke, LNK negatively regulates neural stem cell proliferation, potentially constraining regenerative responses [20]. Furthermore, MEF2A/WWP2-mediated ubiquitination and degradation of LNK have been shown to restore microglial homeostasis and alleviate cerebral microvascular endothelial cell injury, highlighting the importance of LNK regulation in neuroinflammatory processes [21]. Despite its documented roles in neural development, regeneration, and neuroinflammation, the function of LNK in DA neuron survival and its potential involvement in PD pathogenesis remain largely unexplored.

Therefore, we aimed to elucidate the role of LNK in DA neuron degeneration and to define the molecular mechanisms by which LNK influences ferroptotic vulnerability in PD. We examine whether LNK orchestrates a signaling cascade involving the E3 ubiquitin ligase, casitas B-lineage lymphoma (CBL) and the m6A reader, heterogeneous nuclear ribonucleoprotein A2/B1 (HNRPA2B1), ultimately affecting *GPX4 mRNA* stability and neuronal ferroptosis resistance. Additionally, we explore the therapeutic potential of targeting this pathway using both genetic approaches and pharmacological intervention with lifitegrast, a approved lymphocyte function-associated antigen-1 (LFA-1) antagonist. These findings define the LNK–CBL–HNRNPA2B1–GPX4 axis in ferroptotic regulation and support LNK as a potential therapeutic target in PD.

## 2 Materials and methods

### 2.1 Animal studies

All animal procedures were approved by the Institutional Animal Care and Use Committee (IACUC) of Yangzhou University (Protocol No. 202403246), which serves as the designated local authority for animal welfare. The study is reported in accordance with the Animal Research: Reporting of In Vivo Experiments (ARRIVE) guidelines. To mitigate potential confounding effects of hormonal fluctuations and maintain consistency with established models, only male mice were used. Male C57BL/6J mice (8–10 weeks old, 25 ± 2 g) were purchased from the Comparative Medicine Center of Yangzhou University (Yangzhou, China). Global LNK-deficient mice (*Lnk*^*−/−*^) on a C57BL/6J background were a generous gift from Dr. Yu Duonan (Yangzhou University). Lnk floxed (*Lnk*^*f/f*^) and DAT-CreERT2 mice were obtained from Cyagen Biosciences (Suzhou, China). Dopamine transporter (DAT)-specific conditional knockout mice (*DAT*^^(*CreERT2*)^); *Lnk*^^(*flox/flox*)^, hereafter referred to as *LNK*^*ΔDat*^ were generated by crossing these lines. All animals were housed in a specific-pathogen-free (SPF) facility under a 12-h light/dark cycle (lights on at 08:00) with ad libitum access to food and water.

### 2.2 Genotyping

Genomic DNA was extracted from tail biopsies using an alkaline lysis protocol. Briefly, tissue samples were incubated in a buffer containing 25 mM NaOH and 0.2 mM EDTA at 95 °C for 30 min, followed by neutralization with 40 mM Tris-HCl (pH 5.5). Polymerase chain reaction (PCR) was performed with genotype-specific primers (Supplementary Table 1) using GoTaq® Green Master Mix (Promega, Madison, WI, USA). The resulting amplicons were resolved by electrophoresis on a 2 % agarose gel to determine the genotype of each animal.

### 2.3 MPTP-induced PD model and drug administration

To establish a preclinical model of PD, age-matched male mice were administered daily intraperitoneal (i.p.) injections of either MPTP (25 mg/kg body weight; M0896, Sigma-Aldrich, St. Louis, MO, USA) dissolved in saline or an equivalent volume of saline vehicle for 7 consecutive days. For studies involving global knockout mice, animals were randomly assigned to four groups: WT control, *Lnk*^*^−/−^*^ control, WT + MPTP, and *Lnk*^*−/−*^ + MPTP. For conditional knockout studies, *Lnk*^*f/f*^ and *LNK*^*ΔDAT*^ mice were assigned to four groups: *Lnk*^*f/f*^ control, *LNK*^*ΔDAT*^ control, *Lnk*^*f/f*^ + MPTP, and *LNK*^*ΔDAT*^ + MPTP (n = 4–11 mice per group). At the experiment's conclusion, striatal tissue was snap-frozen for metabolomics analysis, and brain hemispheres were processed for immunoblotting, qPCR, or histology.

Continuous intracranial infusion of Lifitegrast was performed using Alzet® micro-osmotic pumps (model 1004, DURECT Corporation) to achieve sustained delivery over 21 days [22,23]. Following completion of MPTP administration, mice underwent stereotaxic surgery under aseptic conditions. A brain infusion cannula (Brain Infusion Kit 3, Alzet) was implanted into the substantia nigra according to stereotaxic coordinates, and connected via PE tubing to a subcutaneously placed micro-osmotic pump prefilled with Lifitegrast solution (500 μM in sterile saline, ultrasonic-assisted dissolution; pump flow rate 0.11 μL/h). The pump reservoir concentration was selected to yield a target steady-state tissue concentration of ∼1 μM based on diffusion and clearance considerations. Wounds were sutured, postoperative analgesia was administered, and animals were monitored daily. Behavioral and histological assessments were performed on day 21 post-implantation unless otherwise indicated.

### 2.4 Behavioral analysis

Motor function was evaluated seven days after the final MPTP injection. Prior to formal testing, all mice underwent a 5-day habituation period to the testing environment to minimize stress-induced variability.

• Open-field test: Each mouse was introduced into the center of a 40 × 40 cm arena. Locomotor activity, including total distance traveled and mean velocity, was automatically tracked for 5 min using SMART 3.0 software (Panlab, Barcelona, Spain).
• Rotarod test: Motor coordination and balance were assessed using an accelerating rotarod apparatus (Ugo Basile, Varese, Italy). Mice were placed on the rod as it accelerated from 4 to 40 rpm over a 300 s period. The latency to fall was recorded for each of three consecutive trials.
• Pole test: To evaluate bradykinesia and motor coordination, mice were positioned facing upward at the top of a vertical wooden pole (50 cm height, 1 cm diameter). The time taken to turn and descend to the cage floor was measured.

### 2.5 Immunohistochemistry and image analysis

Mice were deeply anaesthetized with Avertin (300 mg/kg, i.p.; Sigma-Aldrich,MO, USA) and perfused transcardially with chilled phosphate-buffered saline (PBS), followed by 4 % paraformaldehyde (PFA) in phosphate buffer. Brains were post-fixed in 4 % PFA for 48 h at 4 °C, cryoprotected by sequential immersion in 20 % and 30 % sucrose solutions, and coronally sectioned at 30 μm or 15 μm using a Leica CM1950 cryostat (Leica Biosystems, Wetzlar, Germany). For TH staining, free-floating sections were incubated in 0.6 % H_2_O_2_ in PBS to quench endogenous peroxidases, permeabilized with 0.5 % Triton X-100 in PBS, and blocked with 10 % normal goat serum. Sections were then incubated with primary antibodies (see Supplementary Table 2 for details) overnight at 4 °C, followed by incubation with biotinylated secondary antibodies (1:200; Vector Laboratories, Burlingame, CA, USA) and signal development using the VECTASTAIN Elite ABC kit (Vector Laboratories). DAB-stained sections were digitized using a Pannoramic 250 FLASH slide scanner (3DHISTECH, Budapest, Hungary). Striatal TH-positive fiber density was quantified using Image-Pro Plus 6.0 (Media Cybernetics, Rockville, MD, USA).

### 2.6 Immunofluorescence and confocal microscopy

Tissue sections or cultured cells were treated with 0.2 % Triton X-100 in PBS for 15 min to permeabilize membranes, then blocked with 10 % bovine serum albumin (BSA) in PBS for 1 h at ambient temperature. Sections were exposed overnight at 4 °C to primary antibodies (listed in Supplementary Table 2). Following triple rinses in PBS, samples were incubated for 2 h at ambient temperature in darkness with the respective fluorophore-linked secondary antibodies. Nuclei were counterstained with Hoechst 33342 (1:1000; Invitrogen, Carlsbad, CA, USA), and images were captured using a Leica STELLARIS 5 confocal laser scanning microscope (Leica Microsystems, Wetzlar, Germany). or Leica SP8 laser scanning confocal microscope (Leica Microsystems, Wetzlar, Germany). Mean fluorescence intensity was quantified using Fiji software (ImageJ, v1.53, NIH).

### 2.7 Immunoblotting

Microdissected substantia nigra and striatum tissues or cells were homogenized in ice-cold RIPA buffer (Beyotime Biotechnology, Shanghai, China) supplemented with a protease and phosphatase inhibitor cocktail (MedChemExpress, Monmouth Junction, NJ, USA). Protein concentration was assessed with a BCA assay kit (Proteintech Group, Wuhan, China). Equal amounts of protein (30 μg per lane) were resolved by SDS-PAGE and transferred via electroblotting to PVDF membranes. Membranes were pre-incubated in 5 % non-fat milk in TBST for 1 h, then exposed overnight at 4 °C to primary antibodies (Supplementary Table 2). After washing, they were incubated with HRP-linked secondary antibodies (1:5000) for 1 h. Chemiluminescent signals were detected using the Amersham Imager 600, and band intensities were quantified with ImageJ software (v1.53, NIH**,** MD, USA).

### 2.8 Cell culture, transfection, and treatment

• *Primary Neuronal Culture:* Primary midbrain neurons were prepared from ventral midbrains dissected from E13.5 mouse embryos in ice-cold D-Hanks’ Balanced Salt Solution (HBSS; Solarbio, Beijing, China). Tissues were enzymatically dissociated with 0.25 % Trypsin-EDTA (Gibco, Grand Island, NY, USA) for 15 min at 37 °C, followed by mechanical trituration using fire-polished Pasteur pipettes. Neurons were seeded onto poly-d-lysine-coated surfaces at a density of 1 × 10^6^ cells/well (6-well plates) or 6 × 10^5^ cells/well (coverslips) and cultured in Neurobasal medium supplemented with B27 (Gibco, Grand Island, NY, USA) and GlutaMAX (Gibco, Grand Island, NY, USA). Cultures were maintained at 37 °C in a humidified incubator with 5 % CO_2_, and half-media changes were performed every 3 days.
• *Primary Glial Culture and Microglia Isolation:* Primary mixed glial cultures were established from the whole brains of neonatal (P0–P1) mouse pups. Following the removal of meninges, brain tissues were dissociated using 0.125 % trypsin, and the resulting cell suspension was seeded into poly-d-lysine-coated T25 flasks. Cultures were maintained in Dulbecco's Modified Eagle Medium/Nutrient Mixture F-12 (DMEM/F12; Gibco, Grand Island, NY, USA) supplemented with 10 % fetal bovine serum (FBS; Gibco, Grand Island, NY, USA). At 14–20 days *in vitro* (DIV), microglia were isolated from the mixed glial monolayer by orbital shaking (200 rpm, 4 h). The purity of the isolated microglia was confirmed to be >95 % via immunostaining for Iba1. The remaining adherent cells, predominantly astrocytes, were used for subsequent experiments.
• *Cell Lines and Transfection:* HEK293T cells were purchased from OriCell (Shanghai, China; catalog no. YC-A006; RRID: CVCL_0063). SH-SY5Y human neuroblastoma cells were purchased from OriCell (Shanghai, China; catalog no. YC-D014; RRID: CVCL_0019). Both HEK293T and SH-SY5Y cell lines were maintained in high-glucose Dulbecco's Modified Eagle Medium (Gibco, Grand Island, NY, USA) supplemented with 10 % FBS (Gibco) and 1 % penicillin-streptomycin (MedChemExpress, Monmouth Junction, NJ, USA). Expression plasmids and small interfering RNAs (siRNAs) were sourced from Zebrafish Bio and GenePharma, respectively (sequences provided in Supplementary Table 3). Transient transfections were performed using HighGene Plus Transfection Reagent (ABclonal, Wuhan, China) for HEK293T cells or Lipofectamine 2000 (Thermo Fisher Scientific, Waltham, MA, USA) for SH-SY5Y cells, following the manufacturers' protocols.
• *MPP* ^*+*^ *Treatment:* For the induction of cellular neurotoxicity, cells cultured to approximately 80 % confluency were exposed to 1 mM 1-methyl-4-phenylpyridinium (MPP^+^; Sigma-Aldrich, St. Louis, MO, USA) in complete culture medium for 24 h.
• *Lifitegrast Treatment:*To investigate the protective effects of Lifitegrast in a cellular model of PD, SH-SY5Y cells were first subjected to neurotoxic stress by treatment with 1 mM MPP^+^ for 24 h. Subsequently, the medium was replaced, and cells were incubated with either 1 μM Lifitegrast (MedChemExpress, Monmouth Junction, NJ, USA; HY-17524) or a corresponding H_2_O vehicle control for an additional 24 h. Following this treatment period, a series of analyses were performed.

### 2.9 Quantitative real-time PCR (RT-qPCR)

Total RNA was extracted from cultured cells or brain tissue using the NcmSpin RNA Kit (NCM Biotech, Suzhou, China) per the manufacturer's instructions. Two micrograms of RNA were reverse-transcribed into cDNA using the NCMScript All-in-One RT Premix with dsDNase (NCM Biotech, Suzhou, China). Quantitative PCR was carried out in triplicate for each sample on a QuantStudio 5 Real-Time PCR System (Thermo Fisher Scientific, Waltham, MA, USA) using UltraSYBR Mixture (CWBIO, Beijing, China). The cycling profile comprised initial denaturation at 95 °C for 10 min, followed by 40 cycles at 95 °C for 15 s and 60 °C for 1 min. Relative mRNA levels were calculated using the 2^−ΔΔCt^ method and normalized to β-actin. All primer sequences are listed in Supplementary Table 3.

### 2.10 LC-MS/MS proteomic analysis

For proteomic profiling, protein samples were quantified using a BCA Protein Assay Kit (Beyotime, Shanghai, China), and 40 μg of protein per sample was resolved by 10 % SDS-PAGE and stained with Coomassie Brilliant Blue G-250 (Beyotime, Shanghai, China). Entire gel lanes were excised into 1 mm^3^ pieces for in-gel tryptic digestion following reduction with 10 mM DTT (Sigma-Aldrich, St. Louis, MO, USA) and alkylation with 55 mM iodoacetamide (Sigma-Aldrich, St. Louis, MO, USA). LC-MS/MS analysis was performed using an EASY-nLC 1200 system coupled to a Q-Exactive HF-X mass spectrometer (Thermo Fisher Scientific, Waltham, MA, USA) equipped with a C18 column (75 μm × 25 cm; Agilent Technologies, Santa Clara, CA, USA). Peptide identification and quantification were conducted using MaxQuant software (v1.6.17; Max Planck Institute, Munich, Germany) with Mascot search engine (v2.2; Matrix Science, London, UK) against the UniProtKB/Swiss-Prot *Mus musculus* database. Proteins identified with ≥2 unique peptides and FDR <1 % were considered for downstream analysis.

### 2.11 Protein Interaction Assays

• *Immunoprecipitation (IP) and In Vivo Ubiquitination Assays:* Cells were lysed in ice-cold IP buffer [150 mM NaCl, 10 mM Tris-HCl (pH 7.4), 1 mM EDTA, 1 % NP-40] supplemented with a protease and phosphatase inhibitor cocktail. Lysates were pre-cleared with Protein A/G Magnetic Beads (MedChemExpress, Monmouth Junction, NJ, USA) for 1 h at 4 °C. The pre-cleared supernatant was then incubated with target-specific antibodies or control IgG overnight at 4 °C, followed by incubation with fresh Protein A/G Magnetic Beads for an additional 4 h. The beads were washed extensively with IP buffer, and bound proteins were eluted by boiling in SDS loading buffer. For *in vivo* ubiquitination assays, cells were pre-treated with 10 μM MG132 (MedChemExpress, Monmouth Junction, NJ, USA) for 6 h prior to harvest. Lysis was performed under denaturing conditions in a buffer containing 1 % SDS. The lysates were then sonicated, heated at 95 °C for 10 min, and diluted 10-fold with non-denaturing IP buffer before proceeding with the immunoprecipitation as described above.
• *GST Pull-Down Assay:* Recombinant GST-tagged proteins and GST alone (as a control) were expressed in *Escherichia coli* BL21(DE3) and purified using GST resin (Beyotime, Shanghai, China). Purified proteins were immobilized on MagneGST beads (Promega, Madison, WI, USA) and incubated with pre-cleared cell lysates overnight at 4 °C. After extensive washing, bound proteins were eluted with glutathione buffer and analyzed by immunoblotting.
• *Biolayer Interferometry (BLI):* Binding kinetics were measured using an Octet RED96 system (Sartorius, Göttingen, Germany). Biotinylated target proteins were loaded onto Super Streptavidin (SSA) biosensors (Sartorius, Göttingen, Germany). biosensors. The association of analytes and their subsequent dissociation were monitored in real-time in PBST buffer [PBS with 0.02 % Tween-20; Sigma-Aldrich, St. Louis, MO, USA]. Kinetic parameters (K_a_, Kᴅ, Kᴱ) were fitted to a 1:1 binding model using Octet Data Analysis Software (Sartorius, Göttingen, Germany).
• *MicroScale Thermophoresis (MST):* Binding affinity between LNK and Lifitegrast was quantified by MST using Monolith NT.115 instrument (NanoTemper Technologies, Munich, Germany). Recombinant human LNK protein (Sino Biological, Beijing, China; catalog no. 12104-H08H) was fluorescently labeled with the Monolith His-Tag Labeling Kit RED-tris-NTA (NanoTemper Technologies, Munich, Germany, MO-L018). For the binding assay, labeled LNK (50 nM) was incubated with a 16-point serial dilution of Lifitegrast (MCE, HY-17524; ranging from 100 μM to 3.05 nM) in assay buffer [20 mM HEPES pH 7.4, 150 mM NaCl, 0.05 % Tween-20]. After a 10-min incubation at room temperature, samples were loaded into standard capillaries and analyzed on a Monolith NT.115 instrument (NanoTemper Technologies, Munich, Germany) at 25 °C (40 % LED power, medium MST power). The equilibrium dissociation constant (Kd) was derived by fitting the normalized fluorescence data using MO.Affinity Analysis Software (NanoTemper Technologies, Munich, Germany). *2.*12 RNA*-Protein Interaction Assays*
• *RNA Immunoprecipitation (RIP):* RIP assays were performed using the EZ-Magna RIP Kit (Millipore, Burlington, MA, USA). Cell lysates were incubated with magnetic beads pre-coated with the antibody of interest or a non-specific IgG. After washing, co-precipitated RNA was purified and quantified by RT-qPCR. Enrichment was calculated as fold-change over the IgG control, normalized to input.
• *Methylated RNA Immunoprecipitation (MeRIP):* MeRIP was conducted with the Magna MeRIP™ m^6^A Kit (Millipore, Burlington, MA, USA). Total RNA was fragmented and immunoprecipitated with an anti-m^6^A antibody or control IgG. The abundance of m^6^A-modified RNA was determined by RT-qPCR and presented as fold enrichment relative to input.

### 2.12 Subcellular fractionation

Nuclear and cytoplasmic fractions were prepared from cultured cells using a Nuclear and Cytoplasmic Protein Extraction Kit (Beyotime, Shanghai, China; P0027). The purity of each fraction was rigorously validated by immunoblotting for compartment-specific markers (β-actin for cytoplasm, Histone H3 for nucleus).

### 2.13 Flow cytometry

Data were acquired using a CytoFLEX LX flow cytometer (Beckman Coulter, Brea, CA, USA) and analyzed with CytExpert software (Beckman Coulter, Brea, CA, USA); at least 10,000 events/sample.

Cells were stained for 30 min at 37 °C in the dark using specific fluorescent probes:

• Lipid Peroxidation: 10 μM Liperfluo (Dojindo, Kumamoto, Japan)
•Reactive Oxygen Species (ROS): 10 μM 2′,7′-DCFH-DA (Sigma-Aldrich, St. Louis, MO, USA)

### 2.14 Biochemical assays

All biochemical parameters were quantified using commercially available kits following the manufacturers’ instructions. Absorbance or fluorescence was measured on a microplate reader.

• *Lipid Peroxidation (MDA) Assay:* The concentration of malondialdehyde (MDA), an end product of lipid peroxidation, was determined using a colorimetric MDA assay kit (Beyotime, Shanghai, China; S0131S).
•*Glutathione (GSH) Assay:* The ratio of reduced to oxidized glutathione (GSH/GSSG) was quantified using a GSH and GSSG Assay Kit (Beyotime, Shanghai, China; S0053).

### 2.15 Transmission electron microscopy (TEM)

Mice were anaesthetized and perfused with chilled PBS followed by a fixative containing 2 % PFA and 2.5 % glutaraldehyde in 0.1 M phosphate buffer. Cell samples were post-fixed in 1 % osmium tetroxide, dehydrated in a graded ethanol series, and embedded in Epon 812 resin. Ultrathin sections (70 nm) were stained with uranyl acetate and lead citrate, then examined with a JEM-1230 transmission electron microscope (JEOL, Tokyo, Japan).

### 2.16 Metabolomics and lipidomics analyses

•*Metabolomics* Analysis*:* Brain tissue (50 mg) was homogenized in 800 μL methanol:acetonitrile (1:1, v/v; Agilent Technologies, Santa Clara, CA, USA), centrifuged (14,000×*g*, 20 min, 4 °C), vacuum-dried, and reconstituted in 100 μL acetonitrile:water (1:1, v/v; Agilent Technologies, Santa Clara, CA, USA). Quality control samples were analyzed every 5 injections. Untargeted metabolomics was performed using UHPLC-Orbitrap Exploris 480 MS (Thermo Fisher Scientific, Waltham, MA, USA) with ACQUITY BEH Amide column (2.1 × 100 mm, 1.7 μm) in positive/negative ESI modes. Data were processed using XCMS and CAMERA software (v3.0.2). Statistical analysis employed PCA and OPLS-DA with 7-fold cross-validation (R package 'ropls'). Differentially abundant metabolites (VIP >1, P < 0.05) were mapped to KEGG pathways.
• *Lipid*omics: Lipid extraction was performed using modified Bligh-Dyer method with chloroform:methanol (2:1, v/v; Sigma-Aldrich, St. Louis, MO, USA). Lipidomic analysis was conducted using an Agilent 1290 UHPLC system (Agilent Technologies, Santa Clara, CA, USA) coupled to a Bruker Impact II QTOF mass spectrometer (Bruker Daltonics, Billerica, MA, USA). Lipids were separated on a Kinetex C18 column (2.1 × 100 mm, 1.7 μm; Phenomenex, Torrance, CA, USA) and detected in both positive and negative electrospray ionization (ESI) modes with data-dependent MS/MS acquisition. Lipid identification and quantification were performed using LipidSearch software (v4.2; Thermo Fisher Scientific, Waltham, MA, USA) with mass tolerance <5 ppm and retention time window ± 0.5 min. Lipid species with CV < 30 % in QC samples were retained for analysis

### 2.17 Cellular thermal shift assay (CESTA)

To confirm the intracellular engagement of Lifitegrast with LNK, a Cellular Thermal Shift Assay (CESTA) was performed. SH-SY5Y cells were incubated with 250 μM Lifitegrast (HY -17524; MedChemExpress, Monmouth Junction, NJ, USA) or a corresponding H_2_O vehicle for 4 h. Following treatment, cells were lysed in a non-denaturing buffer using repeated freeze-thaw cycles. The cleared lysates were subjected to a thermal gradient for 3 min to induce protein denaturation, followed by high-speed centrifugation (20,000×*g*, 20 min, 4 °C) to pellet the aggregated proteins. The supernatant, containing the soluble protein fraction, was collected for analysis. The amount of soluble LNK remaining was quantified by immunoblotting.

### 2.18 Computational modeling of protein-protein interactions

• *Protein–Protein Docking: CBL and HNRPA2B1*
The predicted three-dimensional structures of CBL and HNRPA2B1 were obtained using AlphaFold (v3.0; DeepMind Technologies, London, UK). Molecular docking simulations were conducted on the HDOCK server (v1.1; Zhang Laboratory, University of Michigan, Ann Arbor, MI, USA) with CBL as the receptor and HNRPA2B1 as the ligand. Docking parameters were set to default values, and the top-ranked conformation based on docking score was selected. The structural complex was visualized in PyMOL (v2.6.0; Schrödinger LLC, New York, NY, USA), focusing on hydrogen bonding and hydrophobic interaction networks at the predicted binding interface.
• *Protein–Protein Docking: LNK and CBL (TKB domain)*
The docking model between LNK and the tyrosine kinase binding (TKB) domain of CBL (residues 1–357) was generated using AlphaFold3 with CBL modeled in its phosphorylated state. Molecular docking was performed on the HDOCK server, designating LNK as the ligand and the CBL TKB domain as the receptor. Standard docking parameters were used, and the highest-scoring model was selected for further analysis. PyMOL visualization was used to map key intermolecular contacts, including hydrogen bonds and electrostatic interactions, between LNK and phosphorylated residues within the CBL TKB domain.

### 2.19 Structure-based virtual screening targeting the LNK SH2 domain

Structure-based virtual screening of small compounds was carried out using the MTiOpenScreen web server (https://bioserv.rpbs.univ-paris-diderot.fr/services/MTiOpenScreen/) [24]. The crystal structure of the LNK SH2 domain was obtained from the PDB database (PDB ID: 7R8W). For the virtual screening, the structure of the LNK SH2 domain was uploaded, and small compounds were selected from the drug-lib database. In the virtual screening process, the “list of residues” mode was employed for grid calculation. Specifically, eight residues of the LNK SH2 binding sites were input as follows: '_A_SER_368__, _A_SER_366__, _A_ARG_364__, _A_ARG_343__, _A_GLN_399__, _A_LYS_384__, _A_PRO_419__, _A_GLU_421__'. Structural visualizations were generated using MOE2019.01 software and UCSF ChimeraX.

### 2.20 Statistical analysis

All statistical analyses were performed using GraphPad Prism 9.0 (GraphPad Software). Data normality was assessed using the Shapiro–Wilk test. For comparisons between two groups, two-tailed unpaired Student's t-tests were used. For comparisons among three or more groups, one-way analysis of variance (ANOVA) followed by Tukey's post-hoc test for multiple comparisons was employed. Data are expressed as mean ± SEM from at least three independent experiments. The number of biological replicates (n) for each experiment is specified in the corresponding figure legends.

A P-value <0.05 was considered statistically significant.

## 3 Results

### 3.1 LNK deficiency attenuates MPTP-induced neuropathology

**Fig. 1:**
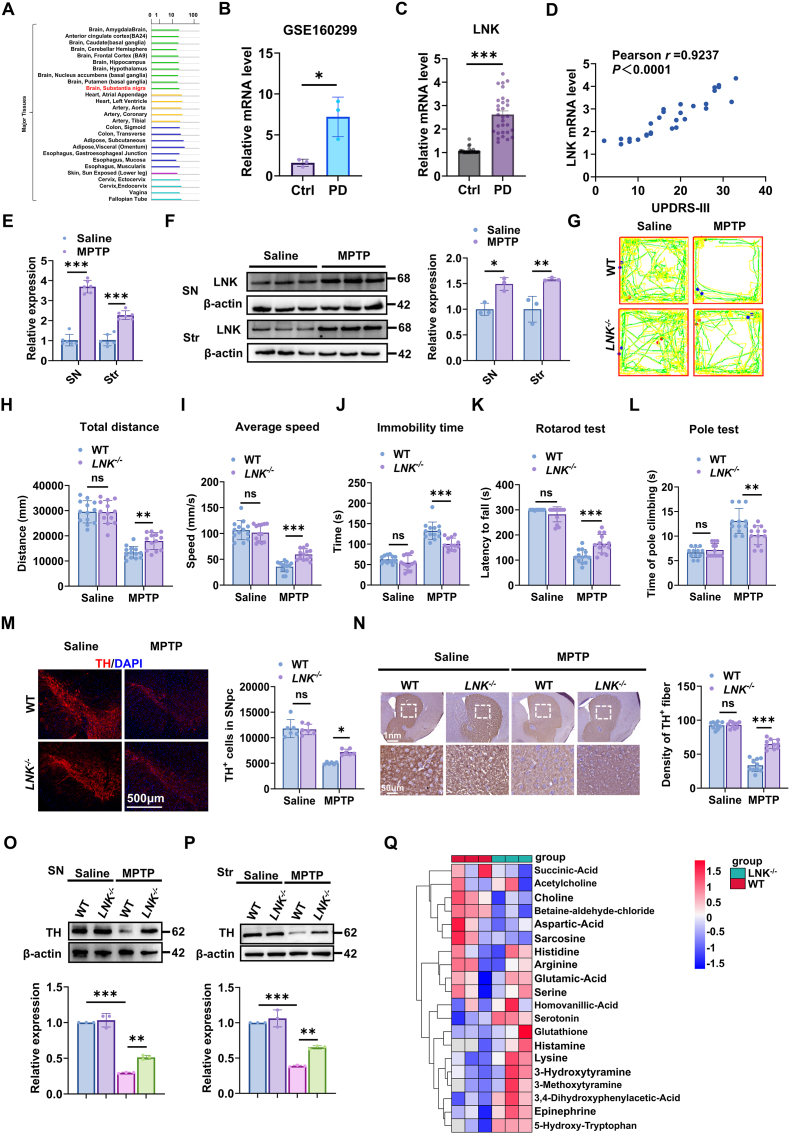
***Lnk* deficiency ameliorates MPTP-induced motor deficits and nigrostriatal neurodegeneration**. **(A)** Expression profile of LNK in the human brain. Data retrieved from a public database (e.g., GTEx Portal). **(B)** LNK mRNA levels in peripheral blood from PD patients and healthy controls, based on analysis of GSE160299. **(C)** Relative mRNA expression of *LNK* in peripheral blood from PD patients (n = 30) and healthy controls (n = 30). **(D)** Pearson correlation analysis between peripheral blood *LNK* mRNA levels and UPDRS-III motor scores in PD patients (n = 30). **(E)** RT-qPCR analysis of *Lnk* mRNA expression in the SN and striatum of C57BL/6 mice 7 days after the final injection of saline or MPTP (n = 6). **(F)** Representative immunoblots and quantification of LNK protein levels in the SN and striatum of C57BL/6 mice. β-actin served as the loading control (n = 3). **(G**–**J)** Open-field test assessing locomotor activity. Representative traces (G) and quantification of total distance traveled (H), average speed (I), and immobility time (J) for WT and *Lnk*^−/−^ mice treated with saline or MPTP (n = 12). **(K)** Latency to fall in the accelerating rotarod test, evaluating motor coordination (n = 12). **(L)** Time to descend in the pole test, assessing bradykinesia (n = 12). **(M)** Representative immunofluorescence images of tyrosine hydroxylase (TH, red) in the SNpc. Right: Quantification of TH-positive cells (n = 6). Scale bar, 500 μm. **(N)** Representative immunohistochemistry images of TH-positive fibers in the striatum. Lower panels are magnified views of the boxed regions. Right: Quantification of the optical density (O.D.) of TH fibers (n = 10). Scale bars, 1 mm (top) and 50 μm (bottom). **(O, P)** Representative immunoblots and quantification of TH protein levels in the SN (O) and striatum (P) from the indicated groups (n = 3). **(Q)** Rank plot from targeted metabolomics illustrating differentially abundant striatal metabolites between MPTP-treated WT and *Lnk*^−/−^ mice (n = 3). Data are presented as mean ± SEM. Statistical significance was determined by two-tailed unpaired Student's t-test (B, C), Pearson correlation (D), or two-way ANOVA with Tukey's post-hoc test (E, F, H, I, J, K, L, M, N, O, P). ∗*P* < 0.05, ∗∗*P* < 0.01, ∗∗∗*P* < 0.001. ns, not significant.

Analysis of the Genotype-Tissue Expression (GTEx) dataset revealed detectable LNK expression in the human SN (Fig. 1A). Similarly, analysis of the GSE160299 dataset demonstrated significantly elevated LNK mRNA levels in the peripheral blood of individuals with PD compared with healthy controls (Fig. 1B). To further validate the clinical relevance of this finding, we quantified LNK mRNA levels in peripheral blood samples from a cohort of patients with PD. Consistent with the public dataset, *LNK* expression was significantly upregulated (Fig. 1C) and exhibited a positive correlation with motor symptom severity, as measured by the UPDRS-III score (Fig. 1D). To determine whether this upregulation is recapitulated in an experimental model of PD, we administered MPTP to C57BL/6 mice. Consistent with the human data, reverse transcription quantitative polymerase chain reaction (RT-qPCR) analysis revealed significantly increased *LNK* mRNA levels in both the SN and striatum of MPTP-treated mice compared with saline-treated controls (Fig. 1E). This transcriptional increase was accompanied by a corresponding elevation in LNK protein levels in these regions, as confirmed by immunoblotting (Fig. 1F).

To investigate the functional role of LNK in PD pathogenesis, we subjected *LNK*-deficient (*LNK*^−/−^) and wild-type (WT) littermates to MPTP administration and assessed their motor performance. In the open-field test, MPTP-treated WT mice exhibited pronounced motor impairments, including reduced total distance traveled, decreased average speed, and prolonged immobility time. Notably, these deficits were significantly attenuated in *LNK*^−/−^ mice (Fig. 1G–J). Consistent with these findings, in assessments of motor coordination and bradykinesia, MPTP-treated *LNK*^−/−^ mice exhibited a longer latency to fall in the accelerating rotarod test and a shorter descent time in the pole test compared with WT controls (Fig. 1K and L). Collectively, these behavioral results indicate that the genetic ablation of *LNK* provides substantial protection against MPTP-induced motor dysfunction.

Building on these behavioral findings, we next examined the core neuropathological features of the nigrostriatal pathway. Immunofluorescent staining for tyrosine hydroxylase (TH), the rate-limiting enzyme in dopamine synthesis, revealed a significant loss of TH-positive neurons in the SNpc of MPTP-treated WT mice. This neuronal loss was significantly attenuated in MPTP-treated *LNK*^−/−^ mice (Fig. 1M). Correspondingly, the density of TH-positive nerve terminals in the striatum was substantially preserved in MPTP-treated *LNK*^−/−^ mice (Fig. 1N). These findings were corroborated by immunoblot analyses, which demonstrated significantly higher TH protein levels in both the SN and striatum of MPTP-treated *LNK*^−/−^ mice relative to their WT counterparts (Fig. 1O and P).

To elucidate the neurochemical mechanisms underlying the protection conferred by LNK deficiency, we conducted targeted metabolomic profiling of striatal tissue. Notably, compared with WT littermates, *LNK*^*−/−*^ mice exhibited a neurochemical signature indicative of attenuated Parkinsonian pathology. Key metabolites of the DA pathway, including 3-hydroxytyramine (dopamine), its metabolite 3-methoxytyramine, and the major catabolite 3,4-dihydroxyphenylacetic acid (DOPAC), were significantly elevated in *LNK*^*−/−*^ mice. Moreover, monoaminergic neurotransmitters such as serotonin, its precursor 5-hydroxytryptophan, and epinephrine were broadly upregulated. Conversely, metabolites associated with the cholinergic pathway and one-carbon metabolism—including choline, betaine aldehyde chloride, sarcosine, and the amino acid neurotransmitter aspartic acid—were significantly reduced (Fig. 1Q). Collectively, these metabolic adaptations indicate that LNK deficiency reshapes the striatal neurochemical landscape following neurotoxin exposure, preserving DA and serotonergic homeostasis and thereby underpinning the observed neuroprotection. We next quantified the expression of key neurotrophic factors in the SNpc using RT-Qpcr [22]. Following MPTP administration, *LNK*^*−/−*^ mice exhibited a significant upregulation of brain-derived neurotrophic factor (*Bdnf*), glial cell line-derived neurotrophic factor (*Gdnf*), insulin-like growth factor 1 (*Igf1*), *nerve growth factor (Ngf)*, and fibroblast growth factor (Fgf), compared with their WT littermates (Fig. S1A). These findings indicate that LNK deficiency enhances neurotrophic factor response under neurotoxic stress conditions.

Finally, we investigated the effects of *LNK* deficiency on additional key pathological hallmarks of PD. The accumulation of α-synuclein phosphorylated at serine 129 (pS129-α-Syn) is a central feature of PD pathology [23,24]. Co-immunofluorescence staining in the SNpc revealed a significant increase in pS129-α-Syn signal within the remaining TH-positive neurons of MPTP-treated WT mice, an effect that was substantially attenuated in *LNK*^−/−^ mice (Fig. S1B). This pattern was consistent with pS129-α-Syn staining in the striatum (Fig. S1C) and was further validated by immunoblot analysis, which confirmed reduced pS129-α-Syn levels in both the SN and striatum of *LNK*^−/−^ mice (Fig. S1D). Given that axonal integrity critically depends on myelin preservation, we assessed the effect of LNK deficiency on MPTP-induced demyelination. Staining for myelin basic protein and Luxol Fast Blue revealed a pronounced loss of myelin in the SNpc of WT mice following MPTP treatment. Notably, this demyelination was largely prevented in *LNK*^*−/−*^ mice (Fig. S1E and F).

Collectively, these findings indicate that elevated LNK expression contributes to PD pathogenesis, whereas its deficiency mitigates MPTP-induced neuropathology in mice.

### 3.2 LNK acts in a DA neuron–autonomous manner to drive neurodegeneration

**Fig. 2:**
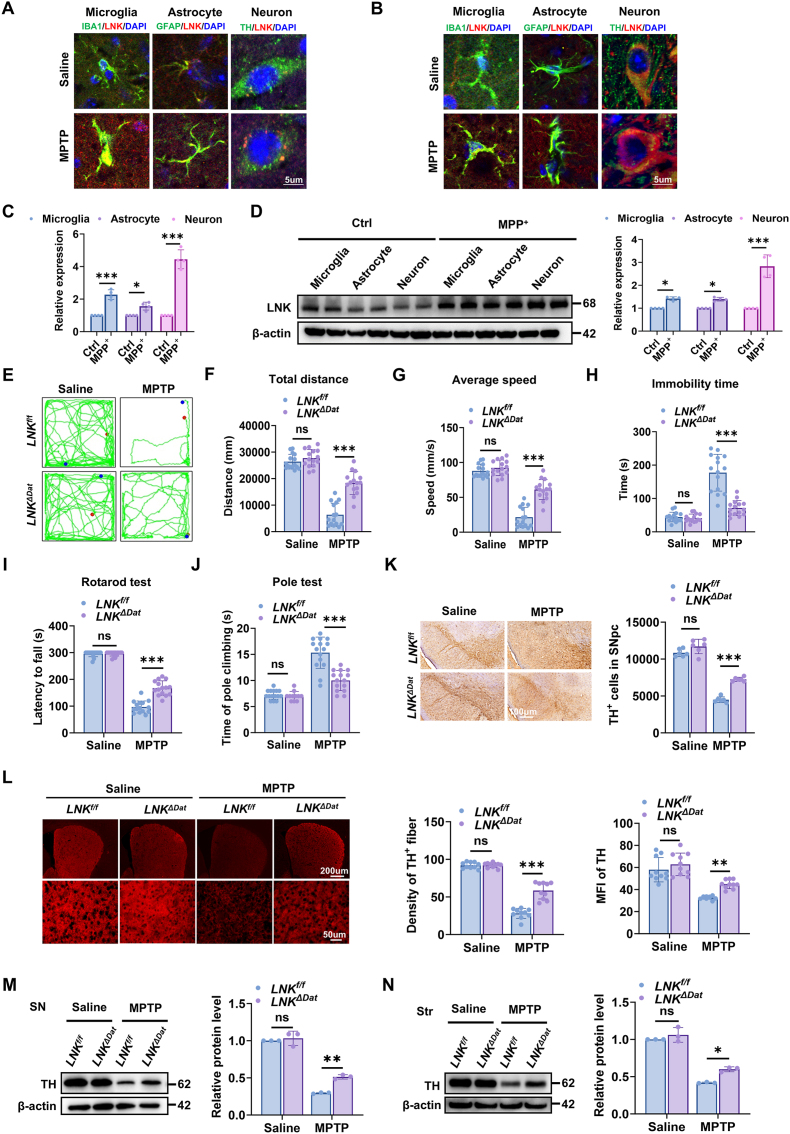
**LNK is preferentially upregulated in dopaminergic neurons and its neuron-specific deficiency confers neuroprotection** **(A)** Fluorescence in situ hybridization (FISH) detection of Lnk mRNA in microglia (IBA1, green), astrocytes (GFAP, green), or dopaminergic neurons (TH, green) in the SNpc of saline- or MPTP-treated C57BL/6 mice. Nuclei were counterstained with DAPI (blue). Scale bar, 5 μm. Full panels with single-channel views are shown in Fig. S2A–C. **(B)** Representative co-immunofluorescence images of LNK (red) with markers for microglia (IBA1, green), astrocytes (GFAP, green), or dopaminergic neurons (TH, green) in the SNpc of saline- or MPTP-treated C57BL/6 mice. Nuclei were counterstained with DAPI (blue). Scale bar, 5 μm. Full panels with single-channel views are shown in Fig. S2E–G. **(C)** Relative mRNA expression of *Lnk* in primary murine microglia, astrocytes, and neurons treated with or without MPP^+^ (1 μM for 24 h), determined by RT-qPCR (n = 4). **(D)** Representative immunoblots and quantification of LNK protein in primary murine microglia, astrocytes, and neurons treated with or without MPP^+^. β-actin served as the loading control (n = 4). **(E**–**H)** Open-field test assessing locomotor activity. Representative traces and quantification of total distance, average speed, and immobility time for *Lnk*^*f/f*^ and *Lnk*^*ΔDat*^ mice treated with saline or MPTP (n = 12). **(I)** Latency to fall in the accelerating rotarod test, evaluating motor coordination (n = 12).(J) Time to descend in the pole test, assessing bradykinesia (n = 12). **(K)** Representative immunofluorescence images of TH (red) in the SNpc of *Lnk*^*f/f*^ and *Lnk*^*ΔDat*^ mice. Right: Quantification of TH-positive cells (n = 6). Scale bar, 500 μm. **(L)** Representative immunofluorescence images of TH (red) in the striatum. Lower panels are magnified views of the boxed regions. Right: Quantification of the density and the MFI of TH fibers (n = 10). Scale bars, 10 μm (top) and 50 μm (bottom). **(M, N)** Representative immunoblots and quantification of TH protein levels in the SN and striatum of *Lnk*^*f/f*^ and *Lnk*^*ΔDat*^ mice (n = 3). Data are presented as mean ± SEM. Statistical significance was determined by one-way ANOVA with Tukey's post-hoc test (C, D) or two-way ANOVA with Tukey's post-hoc test (E− N). ∗P < 0.05, ∗∗P < 0.01, ∗∗∗P < 0.001. ns, not significant.

Having established that systemic LNK deficiency is neuroprotective (Fig. 1, S1), we next examined the cellular distribution of LNK following MPTP administration. Fluorescence in situ hybridization of the SNpc revealed that LNK mRNA was upregulated in microglia (IBA1^+^), astrocytes (GFAP^+^), and DA neurons (TH^+^) compared with saline-treated controls (Fig. 2A, S2A–D). Co-immunofluorescence staining demonstrated corresponding increases in LNK protein across these cell populations (Fig. 2B–S2E–G), and quantification of mean fluorescence intensity (MFI) confirmed the strongest induction in TH^+^ neurons (Fig. S2H). To corroborate these findings *in vitro*, primary murine microglia, astrocytes, and neurons were exposed to the neurotoxin 1-methyl-4-phenylpyridinium‌ (MPP^+^), the active metabolite of MPTP. RT-qPCR analysis revealed that MPP ^+^ treatment elevated *LNK* mRNA expression in all three cell types, with a greater induction observed in neurons (Fig. 2C). Consistently, immunoblot analysis demonstrated a more pronounced increase in LNK protein levels in neurons than in microglia and astrocytes (Fig. 2D). Similarly, human dopaminergic SH-SY5Y neuroblastoma cells exposed to MPP^+^ exhibited significant upregulation of LNK at both the mRNA and protein levels (Fig. S2I and J). Together, these findings indicate that Parkinsonian stressors induce a robust, neuron-predominant upregulation of LNK, suggesting that LNK acts in a dopaminergic neuron–autonomous manner to promote neurodegeneration.

To further delineate the role of DA neuron-derived LNK in the pathological progression of PD, we generated mice with DA neuron–specific LNK deficiency by crossing *LNK*^f/f^ mice with *Dat*-Cre mice (hereafter *LNK*^ΔDat^). Following MPTP administration, we compared the motor performance of *LNK*^ΔDat^ mice with their *LNK*^f/f^ littermates. In the open-field test, *LNK*^ΔDat^ mice displayed significantly greater total distance traveled, higher average speed, and reduced immobility time (Fig. 2E–H). They also demonstrated superior performance in the rotarodand pole tests, showing shorter descent times and longer latency to fall (Fig. 2I and J). These results indicate that DA neuron–specific LNK deficiency significantly mitigates MPTP-induced motor impairments.

Consistently, Immunohistochemistry analysis revealed significant attenuation of TH-positive neuronal loss in the SNpc of MPTP-treated *LNK*^*ΔDat*^ mice compared with MPTP-treated *LNK*^*f/f*^mice (Fig. 2K). Moreover, the integrity of striatal DA terminals—assessed by both fiber density and MFI—was better preserved (Fig. 2L). These findings were further supported by immunoblotting, which showed significantly higher TH protein levels in both the SN and striatum of MPTP-treated *LNK*^*ΔDat*^ mice relative to controls (Fig. 2M and N).

Collectively, these results demonstrate that DA neuron–specific LNK deficiency protects against MPTP-induced DA neurodegeneration and motor dysfunction.

### 3.3 LNK deficiency selectively upregulates the master anti-ferroptotic regulator GPX4

**Fig. 3:**
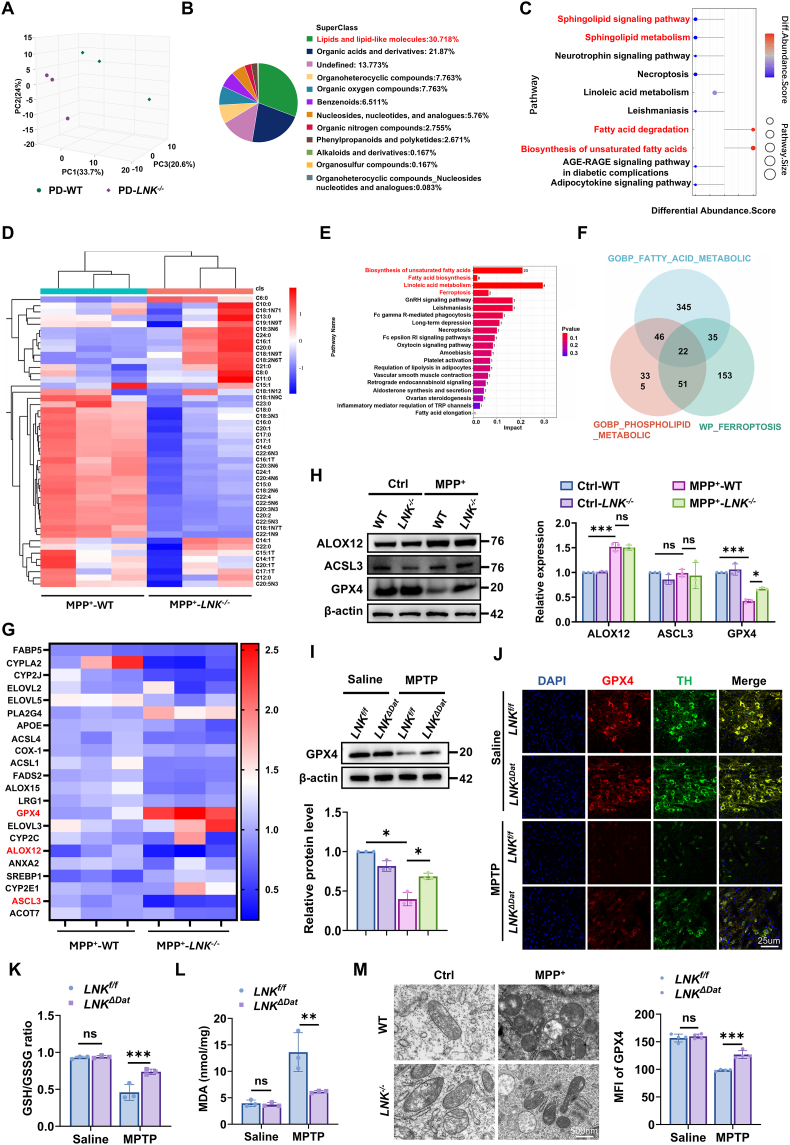
LNK deficiency upregulates GPX4 and attenuates ferroptosis in dopaminergic neurons. **(A)** PCA plot of nigrostriatal metabolomes from WT and *Lnk*^*−/−*^ mice treated with MPTP (n = 3). **(B)** Classification of differentially abundant metabolites identified in (A). **(C)** Kyoto Encyclopedia of Genes and Genomes Pathway (KEGG pathway) enrichment analysis of differential metabolites from (A). Pathways related to lipid metabolism are highlighted. **(D)** Heatmap showing relative abundance of oxidized lipid species in primary WT and *Lnk*^*−/−*^ neurons treated with or without MPP^+^ (1 μM for 24 h), as determined by targeted lipidomics (n = 3). **(E)** KEGG pathway enrichment analysis of differentially expressed genes in *Lnk*^*−/−*^ versus WT primary neurons after MPP^+^ treatment. Bars are ranked by pathway impact factor (x-axis), with bar color indicating the p-value (red denotes higher significance) and the number indicating the gene count. **(F)** Venn diagram depicting the overlap among three functionally annotated gene sets: fatty acid metabolism, phospholipid metabolism, and ferroptosis-related pathways. **(G)** Heatmap of relative mRNA expression for candidate genes from (E) in primary neurons, as measured by RT-qPCR (n = 3). **(H)** Representative immunoblots and quantification of ALOX12, ACSL3, and GPX4 protein levels in primary WT and *Lnk*^*−/−*^ neurons treated with or without MPP^+^ (n = 3). **(I)** Representative immunoblots and quantification of GPX4 protein levels in nigrostriatal tissue from *Lnk*^*f/f*^ and *Lnk*^*ΔDat*^ mice (n = 3). **(J)** Representative co-immunofluorescence images of GPX4 (red), TH (green), and DAPI (blue) in the SNpc of *Lnk*^*f/f*^ and *Lnk*^*ΔDat*^ mice. Bottom: Quantification of GPX4 MFI within TH-positive neurons (n = 4). Scale bar, 20 μm. **(K)** Quantification of the GSH/GSSG ratio in nigrostriatal tissue from MPTP-treated *Lnk*^*f/f*^ and *Lnk*^*ΔDat*^mice (n = 3). **(L)** Quantification of MDA levels in nigrostriatal tissue from MPTP-treated *Lnk*^*f/f*^ and *Lnk*^*ΔDat*^ mice (n = 3). **(M)** Representative TEM images of mitochondria in primary WT and *Lnk*^*−/−*^ neurons treated with or without MPP^+^. Scale bar, 500 nm. Data are presented as mean ± SEM. Statistical significance was determined by two-way ANOVA with Tukey's post-hoc test (H, I, J, K, L). ∗*P* < 0.05, ∗∗*P* < 0.01, ∗∗∗*P* < 0.001. ns, not significant.

Principal component analysis revealed a distinct separation between the metabolic profiles of the two genotypes (Fig. 3A). Lipids and lipid-like molecules represented the largest class of differentially abundant metabolites altered by *LNK* deficiency (Fig. 3B). Consistently, pathway enrichment analysis identified significant perturbations in lipid-associated pathways, including sphingolipid metabolism and fatty acid degradation (Fig. 3C). The most prominent metabolic differences between the two genotypes were concentrated in lipid metabolism pathways. Dysregulated lipid metabolism and the resulting lipid peroxidation are well-established drivers of ferroptosis in DA neurons, a key pathological process in PD [23,24]. To further explore this mechanism, we performed targeted oxidative lipidomic analysis in MPP^+^-treated primary neurons derived from WT and *LNK*^*−/−*^ mice. Upon MPP^+^ exposure, *LNK*-deficient neurons exhibited a significant reduction in the accumulation of multiple oxidized polyunsaturated fatty acid (PUFA)-containing phospholipid species—critical substrates and executors of ferroptosis (Fig. 3D). Pathway enrichment analysis of the differentially expressed genes (DEGs) revealed significant overrepresentation of ferroptosis and multiple lipid metabolism-associated pathways (Fig. 3E). Notably, the biosynthesis of unsaturated fatty acids, fatty acid biosynthesis, and linoleic acid metabolism exhibited high impact scores, indicating substantial perturbations in fatty acid synthesis and remodeling processes [[8], [9], [10]]. The concurrent enrichment of the ferroptosis pathway and lipid metabolic alterations suggests a coordinated disturbance in iron-dependent lipid peroxidation mechanisms. This integrative approach identified 22 high-confidence candidate genes (Fig. 3F). Next, we analyzed the expression of these 22 high-confidence candidate genes at the mRNA level using RT-qPCR. The results revealed that *GPX4*—which encodes the master negative regulator of ferroptosis—exhibited the most marked upregulation, while *Alox12* and *Acsl3* (encoding the pro-ferroptotic enzymes arachidonate 12-lipoxygenase and acyl-CoA synthetase long-chain family member 3, respectively) were significantly downregulated in LNK-deficient neurons at the mRNA level (Fig. 3G). Validation at the protein level confirmed that GPX4 expression was significantly higher in MPP^+^-treated *LNK*^*−/−*^ primary neurons than in MPP^+^-treated WT neurons, whereas ALOX12 and ACSL3 levels remained unchanged (Fig. 3H).

Building on these *in vitro* findings, we next sought to determine whether cell-autonomous deletion of *LNK* in DA neurons was sufficient to induce GPX4 upregulation and mitigate ferroptosis markers *in vivo*. Using our DA neuron-specific knockout model (*LNK*^*ΔDat*^), we subjected mice to MPTP treatment and examined GPX4 expression. Immunoblot analysis of nigrostriatal tissue lysates from MPTP-treated mice revealed a significant increase in total GPX4 protein in *LNK*^*ΔDat*^ mice compared with *LNK*^*f/f*^ controls (Fig. 3I). To assess GPX4 expression specifically in the DA neurons of the SNpc, we performed a co-immunofluorescence staining. Quantitative analysis of MFI showed that GPX4 protein levels were significantly elevated within TH-positive neurons of MPTP-challenged *LNK*^*ΔDat*^ mice relative to their *LNK*^*f/f*^ counterparts (Fig. 3J). This molecular upregulation was associated with functional protection against ferroptosis-induced oxidative stress. Nigrostriatal tissues from MPTP-treated *LNK*^*ΔDat*^ mice exhibited a significantly higher ratio of reduced to oxidized glutathione (GSH/GSSG) and lower levels of the lipid peroxidation marker malondialdehyde (MDA) than did controls (Fig. 3K and L). At the ultrastructural level, transmission electron microscopy (TEM) revealed that while MPP^+^ exposure induced characteristic ferroptotic mitochondrial pathologies in WT neurons—including mitochondrial shrinkage and outer membrane rupture—*LNK*^−/−^ neurons largely maintained normal mitochondrial integrity (Fig. 3M).

Taken together, these results demonstrate that LNK deficiency in DA neurons is associated with upregulation of the ferroptosis suppressor GPX4 and concomitant attenuation of ferroptotic pathology.

### 3.4 LNK promotes ferroptosis by negatively regulating GPX4 in a cellular model of PD

**Fig. 4:**
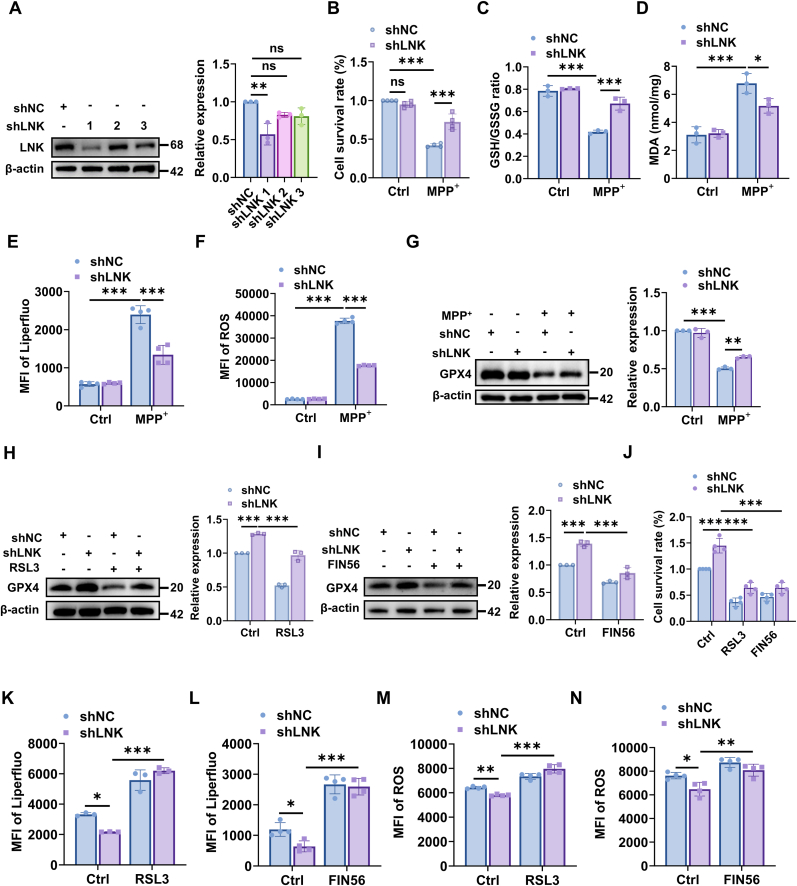
LNK knockdown protects SH-SY5Y cells from MPP^+^-induced ferroptosis by upregulating GPX4 expression All experiments were performed in SH-SY5Y cells stably expressing a non-targeting control shRNA (shNC) or an shRNA against LNK (shLNK). **(A)** Immunoblot and corresponding quantification confirming LNK knockdown efficiency (n = 3). **(B)** Quantification of cell survival (CCK8 assay) in cells treated with or without MPP^+^ (n = 4). **(C)** Quantification of the GSH/GSSG ratio in cells treated with or without MPP^+^ (n = 3). **(D)** Quantification of MDA levels in cells treated with or without MPP^+^ (n = 3). **(E)** Quantification of lipid ROS levels using the Liperfluo probe in cells treated with or without MPP^+^ (n = 4). **(F)** Quantification of general ROS levels using the DHE probe in cells treated with or without MPP^+^ (n = 4). **(G)** Immunoblot and quantification of GPX4 protein in cells treated with or without MPP^+^ (n = 3). **(H)** Immunoblot and quantification of GPX4 after co-treatment with MPP^+^ and RSL3 (100 nM, 6 h) (n = 3). **(I)** Immunoblot and quantification of GPX4 after co-treatment with MPP^+^ and FIN56 (1 μM, 6 h). (n = 3). **(J)** Quantification of cell survival after co-treatment with MPP^+^ and RSL3 or FIN56 (n = 4). **(K)** Quantification of lipid ROS levels after co-treatment with MPP^+^ and RSL3 (n = 3). **(L)** Quantification of lipid ROS levels after co-treatment with MPP^+^ and FIN56 (n = 4). **(M)** Quantification of general ROS levels after co-treatment with MPP^+^ and RSL3 (n = 4). **(N)** Quantification of general ROS levels after co-treatment with MPP^+^ and FIN56 (n = 4). Data are presented as mean ± SEM. Statistical significance was determined by a two-tailed Student's t-test (A) or two-way ANOVA followed by Tukey's post-hoc test (B–N). ∗P < 0.05, ∗∗P < 0.01, ∗∗∗P < 0.001.

Immunoblot analysis confirmed efficient knockdown of endogenous LNK, with shLNK #1 demonstrating the strongest suppression relative to the shNC control (Fig. 4A). Therefore, shLNK #1 was selected for all subsequent loss-of-function experiments. MPP^+^ treatment significantly reduced cell survival in shNC cells, whereas this effect was significantly attenuated in shLNK cells (Fig. 4B). Next, we evaluated canonical hallmarks of ferroptosis. Upon MPP^+^ exposure, LNK knockdown preserved the cellular GSH/GSSG ratio (Fig. 4C) and reduced the accumulation of the lipid peroxidation product MDA (Fig. 4D). It also significantly mitigated the MPP^+^-induced increases in both lipid-specific reactive oxygen species (ROS), measured by Liperfluo (Fig. 4E) and general ROS, measured using dihydroethidium (DHE) (Fig. 4F).

Consistent with a role in ferroptosis regulation, the MPP^+^-induced downregulation of the key ferroptosis suppressor GPX4 was significantly attenuated in shLNK cells (Fig. 4G). To determine whether the neuroprotection conferred by the LNK knockdown was mediated by GPX4, we exposed cells to ferroptosis inducers. Treatment with either the direct GPX4 inhibitor RSL3 or the indirect ferroptosis inducer FIN56 reduced GPX4 protein levels in MPP^+^-treated shLNK cells (Fig. 4H and I) [25,26]. Functionally, RSL3 or FIN56 treatment completely abolished the survival advantage of shLNK cells in the presence of MPP^+^ (Fig. 4J). This loss of protection was accompanied by renewed accumulation of both lipid ROS (Fig. 4K and L) and general ROS (Fig. 4M and N).

Using a complementary gain-of-function approach, we generated SH-SY5Y cells stably overexpressing LNK (oeLNK), as confirmed by western blotting (Fig. S3A). In contrast to LNK knockdown, LNK overexpression exacerbated MPP^+^-induced cell death compared with empty vector-transduced controls (Fig. S3B). Upon MPP^+^ exposure, oeLNK cells also exhibited a more pronounced reduction in the GSH/GSSG ratio (Fig. S3C), greater accumulation of MDA (Fig. S3D), and enhanced generation of both lipid-specific (Fig. S3E) and general ROS (Fig. S3F).

Concurrently, oeLNK cells exhibited a more pronounced downregulation of GPX4 protein following MPP^+^ treatment (Fig. S3G). The MPP^+^-induced suppression of GPX4 expression in oeLNK cells was partially reversed by treatment with the specific ferroptosis inhibitor Liproxstatin-1 (Lip-1) or Ferrostatin-1 (Fer-1) (Fig. S3H and I) [27,28]. Notably, both inhibitors significantly rescued the viability of oeLNK cells from MPP^+^-induced toxicity (Fig. S3J) and reduced the accumulation of both lipid ROS (Fig. S3K and L) and general ROS (Fig. S3M and N).

Collectively, these loss- and gain-of-function experiments identify LNK as a crucial promoter of ferroptosis that negatively regulates GPX4 expression in this cellular model of PD.

### 3.5 LNK post-transcriptionally suppresses GPX4 expression via an m^6^A–HNRPA2B1-dependent mechanism

**Fig. 5:**
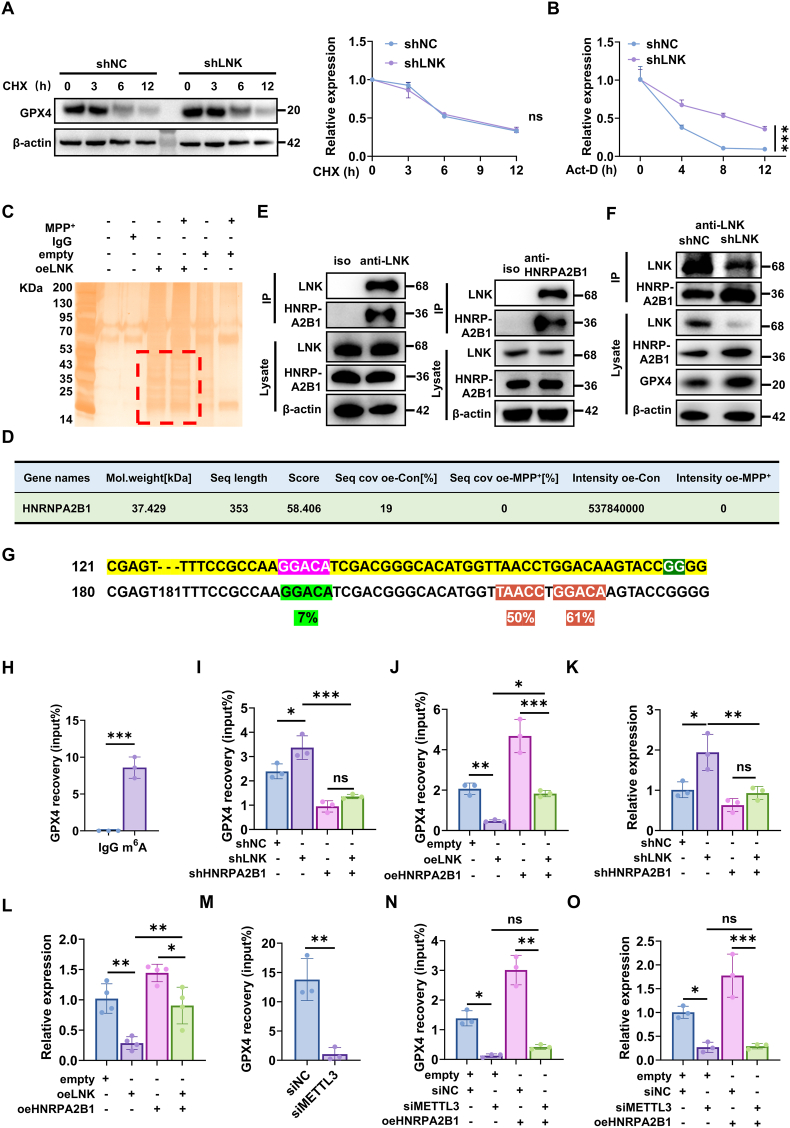
**LNK Negatively Regulates *GPX4* mRNA Stability by Impeding HNRPA2B1-Mediated m^6^A Recognition** SH-SY5Y cells were treated with 1 mM MPP + for 24 h prior to the indicated assays. **(A)** Immunoblot analysis of GPX4 protein stability in shNC and shLNK cells treated with CHX for the indicated durations (n = 3). **(B)** RT-qPCR analysis of *GPX4* mRNA stability in shNC and shLNK cells treated with Act-D for the indicated durations (n = 3). **(C)** Silver stain of proteins co-immunoprecipitated with an anti-LNK antibody from lysates of cells overexpressing LNK. The red box indicates the region excised for mass spectrometry. **(D)** Protein-profile results of HNRPA2B1 in Co-IP/MS analysis. **(E)** Reciprocal endogenous Co-IP in SH-SY5H cells confirming the LNK-HNRPA2B1 interaction. **(F)** Immunoblot of LNK Co-IP assays showing that LNK knockdown attenuates its interaction with HNRPA2B1. Lysates from shNC or shLNK cells were used. **(G)** Schematic of the human *GPX4* 3′ UTR showing the predicted m^6^A consensus motif (RRACH). **(H)** MeRIP-qPCR in MPP^+^-treated cells confirming m^6^A modification of *GPX4* mRNA. Enrichment is shown relative to IgG control (n = 3). **(I)** RIP-qPCR quantifying HNRPA2B1-bound *GPX4* mRNA in MPP ^+^ -treated shNC or shLNK cells with or without concurrent knockdown of HNRPA2B1 (n = 3). **(J)** RIP-qPCR quantifying HNRPA2B1-bound *GPX4* mRNA in MPP ^+^ -treated cells overexpressing an empty vector or LNK, with or without concurrent overexpression of HNRPA2B1 (n = 3). **(K)** RT-qPCR analysis of total *GPX4* mRNA abundance in cells transduced with shNC, shLNK, or shLNK in combination with shHNRPA2B1 (n = 3). **(L)** RT-qPCR analysis of total *GPX4* mRNA abundance in cells overexpressing an empty vector, LNK, or LNK in combination with HNRPA2B1 (n = 4). **(M)** MeRIP-qPCR quantifying HNRPA2B1-bound *GPX4* mRNA in MPP ^+^ -treated cells transfected with siNC or siMETTL3 (n = 3). **(N)** RIP-qPCR quantifying HNRPA2B1-bound *GPX4* mRNA in MPP ^+^ -treated cells co-transfected with siMETTL3 and/or an HNRPA2B1 overexpression plasmid as indicated (n = 3). **(O)** RT-qPCR analysis of total *GPX4* mRNA abundance in cells transfected with control siRNA (siNC), siMETTL3, or siMETTL3 in combination with an HNRPA2B1 overexpression plasmid (n = 3). Data are presented as mean ± SEM. Statistical significance was determined by two-way ANOVA (A, B), a two-tailed Student's t-test (H, M), or one-way ANOVA with Tukey's post-hoc test (I, J, K, L, N, O). ∗P < 0.05, ∗∗P < 0.01, ∗∗∗P < 0.001.

To elucidate the molecular mechanism underlying LNK-mediated repression of GPX4 expression, we first performed cycloheximide (CHX) chase assays to determine whether LNK modulates the rate of GPX4 protein degradation. We found that neither LNK knockdown nor LNK overexpression altered the degradation rate of GPX4 protein (Fig. 5A and S4A). Subsequently, we examined mRNA stability following transcriptional blockade with actinomycin D (Act-D). The results demonstrated that LNK negatively regulates GPX4 mRNA stability. Specifically, the half-life of GPX4 mRNA was significantly prolonged in LNK-knockdown (shLNK) cells but significantly shortened in oeLNK cells (Fig. 5B and S4B). These results establish LNK as a post-transcriptional negative regulator of *GPX4* mRNA stability.

Given that LNK lacks a canonical RNA-binding domain, we hypothesized that it acts as a partner of RNA-binding proteins (RBPs). To identify such interactors, we performed co-immunoprecipitation (Co-IP) using lysates from SH-SY5Y cells overexpressing LNK, with or without MPP+, followed by mass spectrometry (MS) (Fig. 5C). Notably, among the proteins involved in mRNA stability, HNRPA2B1 exhibited the most pronounced reduction in its interaction with LNK after MPP + treatment (Fig. 5D). This physical association was subsequently validated by reciprocal endogenous Co-IP in SH-SY5Y cells (Fig. 5E). Building on these findings, we investigated whether LNK modulates HNRPA2B1 abundance. In SH-SY5Y cells exposed to MPP^+^, LNK knockdown (shLNK) attenuated the MPP^+^-induced reduction of HNRPA2B1, as shown by western blotting (Fig. S4C) and immunofluorescence (Fig. S4D), compared with shNC. Conversely, LNK overexpression (oeLNK) further decreased HNRPA2B1 protein levels under the same conditions relative to empty-vector controls (Fig. S4E and S4F). These results indicate that LNK negatively regulates HNRPA2B1 protein levels under MPP + -induced stress. Finally, we performed Co-IP assays under both LNK knockdown and overexpression conditions to further characterize this interaction. Notably, under MPP^+^-induced stress, the increased co-immunoprecipitation of HNRNPA2B1 observed in LNK-knockdown cells directly paralleled the substantial cellular accumulation of total HNRNPA2B1 and GPX4 proteins (Fig. 5F). In contrast, LNK overexpression under the same conditions resulted in a diminished interaction with HNRPA2B1, coinciding with reduced total levels of HNRPA2B1 and GPX4 proteins (Fig. S4G). HNRPA2B1 is a well-characterized m^6^A reader protein that binds m^6^A-modified transcripts to regulate their stability [29]. Based on this, we hypothesized that LNK modulates GPX4 expression by interfering with the m^6^A-dependent recognition of *GPX4* mRNA by HNRPA2B1. To test this, we first evaluated whether *GPX4* mRNA is a direct m^6^A target. Bioinformatic analysis identified a high-confidence m^6^A modification site in the 3’ UTR of human GPX4, characterized by adjacent adenosine residues with predicted probabilities of 50 % and 61 % within a canonical m^6^A consensus motif (RRACH) (Fig. 5G). Accordingly, primers for qPCR analysis following methylated RNA immunoprecipitation (MeRIP) and RNA immunoprecipitation (RIP) were designed to specifically target the identified m^6^A site. MeRIP-qPCR performed on MPP^+^-treated SH-SY5Y cells confirmed a significant enrichment of *GPX4* mRNA, establishing it as an m^6^A-modified transcript under neurotoxic stress (Fig. 5H). Next, we examined whether LNK modulates the interaction between HNRPA2B1 and *GPX4* mRNA. RIP assay revealed that LNK knockdown significantly enhanced HNRPA2B1 binding to *GPX4* mRNA (Fig. 5I), whereas LNK overexpression significantly suppressed this interaction (Fig. 5J). Consistent with these alterations in RBP binding, total *GPX4* mRNA levels were elevated in shLNK cells and reduced in oeLNK cells following MPP ^+^ treatment (Fig. 5K and L). These effects were HNRPA2B1-dependent: concurrent HNRPA2B1 knockdown abolished both the increased binding and elevated *GPX4* mRNA levels in shLNK cells (Fig. 5I–K), while HNRPA2B1 overexpression partially restored this binding and rescued GPX4 mRNA levels in oeLNK cells (Fig. 5J–L). Actinomycin D chase assays directly confirmed these observations: LNK knockdown enhanced *GPX4* mRNA stability, which was reversed by HNRPA2B1 knockdown (Fig. S4H), whereas LNK overexpression promoted *GPX4* mRNA degradation, which was fully rescued by HNRPA2B1 overexpression (Fig. S4I).

Finally, to confirm that m^6^A modification of the *GPX4* transcript is required for this regulatory mechanism, we knocked down the m^6^A writer enzyme methyltransferase-like 3 (METTL3) in MPP^+^-treated cells. METTL3 knockdown (Fig. S4J) significantly reduced the binding of HNRPA2B1 to *GPX4* mRNA (Fig. 5M). Notably, this loss of interaction could not be rescued by HNRPA2B1 overexpression, indicating an absolute requirement for the m^6^A modification (Fig. 5N). Consequently, *GPX4* mRNA abundance was significantly diminished following METTL3 depletion, regardless of HNRPA2B1 expression levels (Fig. 5O). Together, these findings establish that METTL3-mediated m^6^A modification of *GPX4* mRNA is essential for HNRPA2B1 binding and subsequent transcript stabilization under neurotoxic stress conditions.

**Fig. 6:**
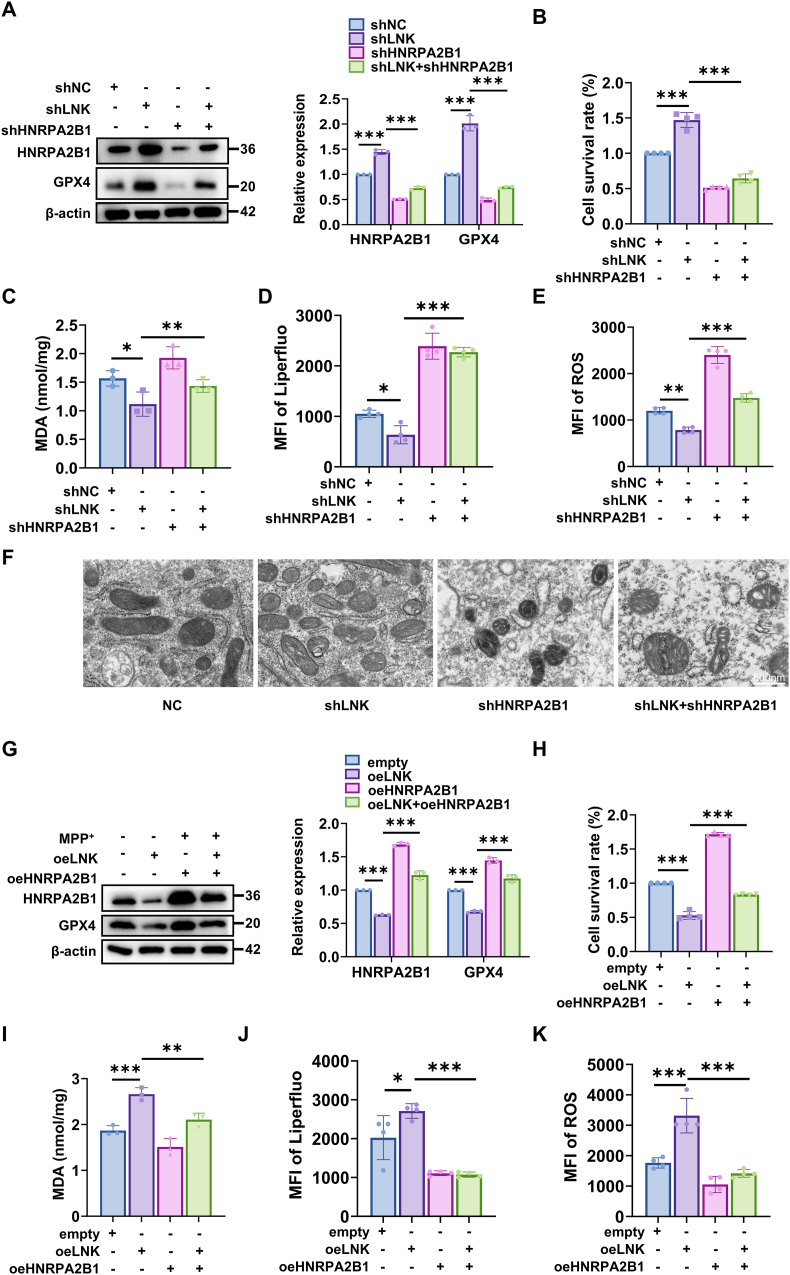
LNK negatively regulates HNRPA2B1 to modulate GPX4-mediated ferroptosis SH-SY5Y cells were treated with 1 mM MPP^+^ for 24 h prior to the indicated assays. **(A)** Immunoblot analysis of HNRPA2B1 and GPX4 protein levels in cells with single (shLNK or shHNRPA2B1) or double knockdown (left), with corresponding quantification (right) (n = 3). **(B)** Quantification of cell viability in the indicated cell groups (n = 4). **(C)** Quantification of MDA levels in the indicated cell groups (n = 3). **(D)** Quantification of lipid ROS levels using the Liperfluo probe in the indicated cell groups. (n = 4). **(E)** Quantification of general ROS levels using the DHE probe in the indicated cell groups. (n = 4). **(F)** Representative TEM images of mitochondrial morphology in the indicated cell groups. Scale bar, 500 nm. **(G)** Immunoblot analysis and corresponding quantification of HNRPA2B1 and GPX4 protein levels in cells with LNK overexpression (oeLNK), HNRPA2B1 overexpression (oeHNRPA2B1), or co-overexpression (n = 3). **(H)** Quantification of cell viability in the indicated cell groups to assess rescue by HNRPA2B1 co-expression (n = 4). **(I)** Quantification of MDA levels in the indicated cell groups to assess rescue by HNRPA2B1 co-expression (n = 3). **(J)** Quantification of lipid ROS levels (Liperfluo) in the indicated cell groups to assess rescue by HNRPA2B1 co-expression (n = 4). **(K)** Quantification of general ROS levels (DHE) in the indicated cell groups to assess rescue by HNRPA2B1 co-expression (n = 4). Data are presented as mean ± SEM. Statistical significance was determined by two-way ANOVA with Tukey's post-hoc test (A, G) or one-way ANOVA with Tukey's post-hoc test (B-E, H–K). ∗*P* < 0.05, ∗∗*P* < 0.01, ∗∗∗*P* < 0.001. ns, not significant.

To determine whether LNK-mediated regulation of ferroptosis depends on HNRPA2B1, we performed a series of knockdown and rescue experiments using an MPP^+^-induced cellular stress model. Quantitative immunoblot analysis revealed that LNK knockdown significantly increased the protein abundance of GPX4, a key negative regulator of ferroptosis. However, this upregulation was substantially attenuated following simultaneous knockdown of HNRPA2B1 (Fig. 6A). Consistently, cellular viability assays demonstrated that while LNK knockdown conferred resistance to MPP^+^-induced cytotoxicity, this protective effect was largely abrogated upon co-knockdown of HNRPA2B1 (Fig. 6B). Parallel assessments of ferroptotic markers revealed that the reductions in MDA, lipid ROS, and general ROS levels conferred by LNK knockdown were similarly reversed by HNRPA2B1 depletion (Fig. 6C–E). TEM further showed that although LNK knockdown preserved mitochondrial integrity, double knockdown cells exhibited shrunken mitochondria with disrupted cristae—a morphological hallmark of ferroptosis (Fig. 6F).

In a complementary gain-of-function approach, LNK overexpression in MPP^+^-treated cells suppressed both HNRPA2B1 and GPX4 protein expression, whereas co-overexpression of HNRPA2B1 partially rescued their expression levels (Fig. 6G). Functionally, the pro-ferroptotic phenotype induced by LNK overexpression—characterized by reduced cell viability and elevated MDA, lipid ROS, and general ROS—was significantly attenuated by HNRPA2B1 co-expression (Fig. 6H–K).

Collectively, these findings reveal that LNK promotes neuronal ferroptosis by disrupting the HNRPA2B1-mediated stabilization of GPX4 mRNA.

### 3.6 LNK mediates proteasomal degradation of HNRPA2B1 via K27-linked polyubiquitination

**Fig. 7:**
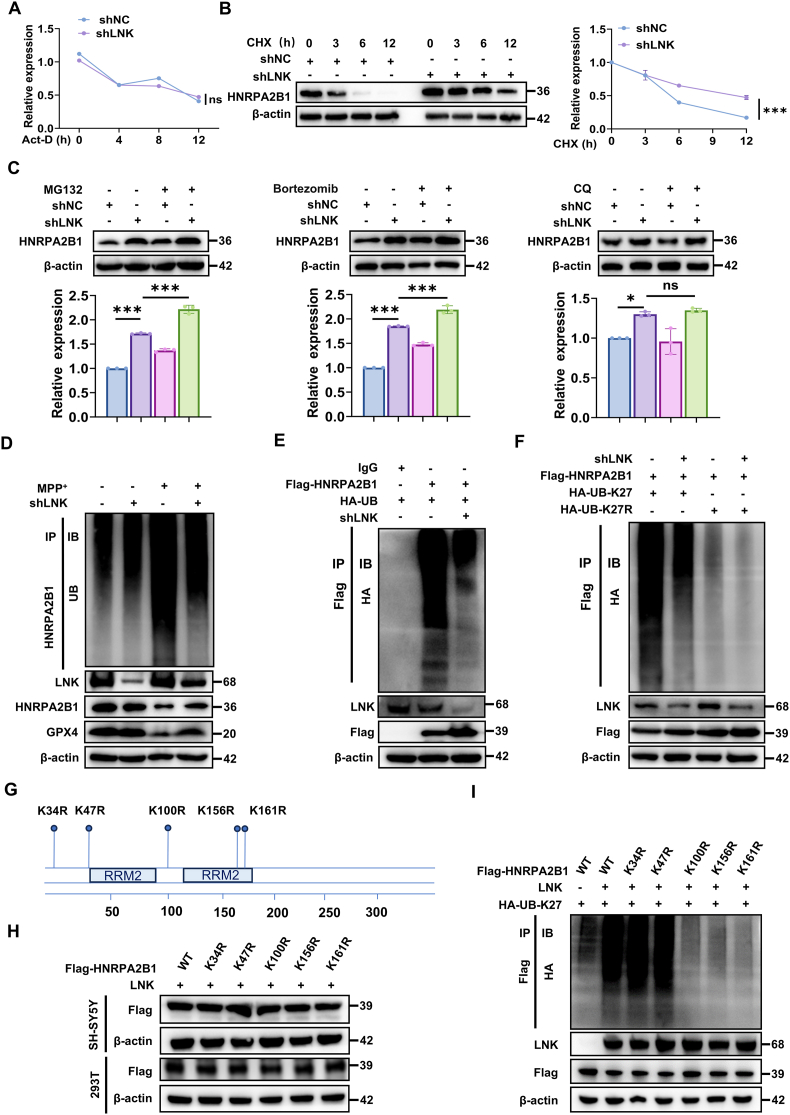
LNK Promotes K27-Linked Polyubiquitination and Proteasomal Degradation of HNRPA2B1 All experiments were performed in SH-SY5Y cells unless otherwise specified. **(A)** RT-qPCR analysis of *HNRPA2B1* mRNA stability in shNC and shLNK cells treated with Act-D (5 μg/mL) under MPP^+^ stress (1 mM, 24 h). n = 3. **(B)** Immunoblot analysis and corresponding quantification of HNRPA2B1 protein stability in shNC and shLNK cells treated with CHX (100 μg/mL) under MPP^+^ stress (n = 3). **(C)** Immunoblot analysis of HNRPA2B1 in shNC and shLNK cells treated with MPP^+^ and co-treated with proteasome inhibitors (MG132, 10 μM; Bortezomib, 100 nM) or a lysosome inhibitor (Chloroquine, CQ; 20 μM). n = 3. **(D)** Analysis of endogenous HNRPA2B1 ubiquitination in SH-SY5Y cells transduced with shNC or shLNK under MPP^+^ stress. Lysates were immunoprecipitated (IP) with an anti-HNRPA2B1 antibody and immunoblotted (IB) with an anti-Ubiquitin antibody. **(E)** Cellular ubiquitination assay in HEK293T cells co-transfected with Flag-HNRPA2B1, HA-Ub, and either shNC or shLNK. Lysates were subjected to IP with an anti-Flag antibody, followed by IB with an anti-HA antibody. **(F)** Cellular ubiquitination assay confirming K27-specific linkage. Cells were co-transfected with Flag-HNRPA2B1, shLNK, and either a ubiquitin construct permitting only K27 linkage (HA-Ub-K27) or its K27R mutant. **(G)** Schematic of the HNRPA2B1 protein, highlighting predicted ubiquitination sites (blue) and the RNA recognition motif (RRM) domains. **(H)** Immunoblot analysis confirming comparable expression of Flag-tagged HNRPA2B1 WT and its K-to-R mutants in SH-SY5Y and HEK293T cells. **(I)** Cellular ubiquitination assay in HEK293T cells to map ubiquitination acceptor sites. Cells were co-transfected with an LNK expression vector (oeLNK), a K27-only ubiquitin construct (HA-Ub-K27), and either WT or mutant Flag-HNRPA2B1 constructs. Data are presented as mean ± SEM. Statistical significance was determined by two-way ANOVA (A, B) or one-way ANOVA with Tukey's post-hoc test (C). ∗∗∗P < 0.001. ns, not significant.

We next investigated whether LNK regulates HNRPA2B1 expression at the transcriptional or post-translational level. LNK knockdown or overexpression did not affect the mRNA decay rate of HNRPA2B1 (Fig. 7A and S5A). However, LNK knockdown significantly prolonged HNRPA2B1 protein half-life (Fig. 7B), whereas LNK overexpression markedly shortened it (Fig. S5B). These results indicate that LNK governs HNRPA2B1 abundance via protein degradation.

To identify the degradation pathway, pharmacological inhibitors were employed. In LNK-overexpressing cells, the reduction in HNRPA2B1 was reversed by proteasome inhibitors MG132 and bortezomib, but not by the lysosome inhibitor chloroquine (CQ) (Fig. S5C). This effect was further confirmed in LNK-knockdown cells (Fig. 7C). Collectively, these results demonstrate that LNK targets HNRPA2B1 for degradation through the ubiquitin-proteasome system.

This finding prompted us to examine HNRPA2B1 ubiquitination. In SH-SY5Y cells exposed to MPP^+^, LNK knockdown significantly reduced the polyubiquitination of endogenous HNRPA2B1 (Fig. 7D). To validate this observation in an exogenous system, we conducted a similar assay in HEK293T cells and found that LNK knockdown reduced the polyubiquitination of co-transfected Flag-HNRPA2B1 (Fig. 7E). Conversely, LNK overexpression enhanced the polyubiquitination of both endogenous and exogenous HNRPA2B1 (Fig. S5D and E), indicating that LNK actively promotes HNRPA2B1 polyubiquitination.

We then examined the specific linkage type of the polyubiquitin chains mediating this effect. Previous studies have implicated K27, K48, and K63 linkages as principal determinants for HNRPA2B1 [[30], [31], [32], [33]]. To dissect the contribution of each linkage, we performed cell-based ubiquitination assays using a panel of ubiquitin constructs: single-lysine constructs (K27-only, K48-only, or K63-only) and lysine-to-arginine point mutations (K27R, K48R, or K63R). In the K-only constructs, all lysine residues except the specified residue were replaced with arginine, allowing the formation of only one type of polyubiquitin chain. In contrast, the point mutants specifically prevented the formation of the respective linkage types. These assays revealed that LNK-induced polyubiquitination of HNRPA2B1 was predominantly mediated by K27-linked chains, with minimal contributions from K48 or K63 linkages (Fig. S5F–H). Notably, the reduction in HNRPA2B1 polyubiquitination observed upon LNK knockdown was dependent on the K27 residue of ubiquitin, as this effect was abolished by the K27R mutation (Fig. 7F). To identify the lysine residues in HNRPA2B1 targeted for ubiquitination, we first predicted potential sites using bioinformatics tools and subsequently generated Flag-tagged HNRPA2B1 constructs with individual lysine-to-arginine (K-to-R) mutations at high-probability sites (Fig. 7G). Western blot analysis confirmed that the basal expression levels of these HNRPA2B1 mutants were comparable to those of the WT HNRPA2B1 (Fig. 7H). We co-expressed these constructs with LNK and HA-tagged K27-only ubiquitin in HEK293T cells. Cellular ubiquitination assays demonstrated that individual mutations at K100, K156, or K161 significantly attenuated LNK-mediated polyubiquitination of HNRPA2B1, whereas mutations at K34 or K47 had no discernible effect (Fig. 7I).

Collectively, these findings delineate a mechanism by which LNK promotes proteasomal degradation of HNRPA2B1. This process is mediated by K27-linked polyubiquitin chain formation, with K100, K156, and K161 identified as critical acceptor sites.

### 3.7 LNK recruits the E3 ligase CBL to mediate K27-linked polyubiquitination of HNRPA2B1

Given that LNK is an adaptor protein lacking intrinsic enzymatic activity, we sought to identify the E3 ubiquitin ligase responsible for LNK-mediated HNRPA2B1 degradation [21,[34], [35], [36]]. We investigated whether CBL, a known LNK-interacting E3 ligase, mediates this process by targeting HNRPA2B1. To assess the structural feasibility of a direct interaction, we performed computational modeling. Molecular docking simulations using protein structures predicted by AlphaFold3 indicated a stable binding interface between CBL and HNRPA2B1, characterized by extensive hydrogen bonding and hydrophobic interactions (Fig. S6A).

**Fig. 8:**
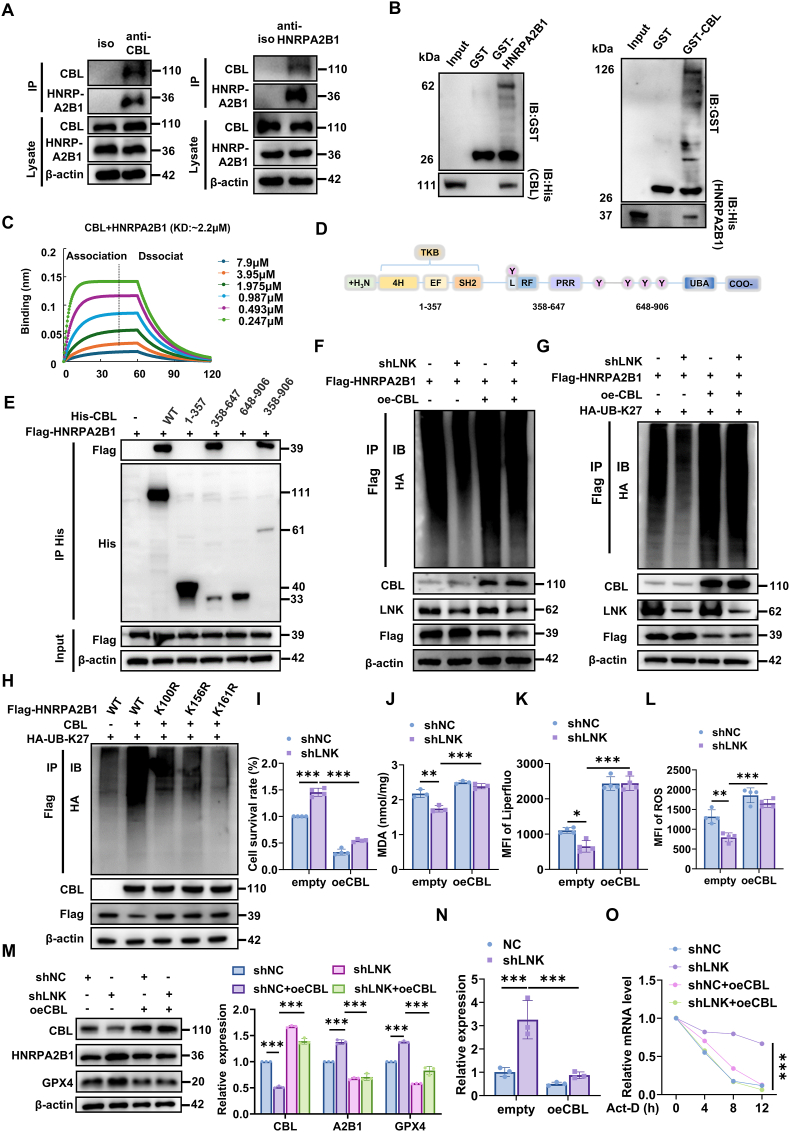
**LNK facilitates CBL-mediated, K27-linked ubiquitination and degradation of HNRPA2B1** **(A)** Reciprocal endogenous co-IP assays in HEK293T cells showing the interactions between CBL and HNRPA2B1. Isotype IgG served as a negative control. **(B)** In vitro GST pull-down assays confirming a direct interaction between purified recombinant HNRPA2B1 and CBL proteins. **(C)** BLI sensorgram measuring the binding kinetics between purified CBL and HNRPA2B1 proteins. The calculated Kᴅ is shown. **(D)** Schematic diagram of full-length human CBL mutants. TKB, Tyrosine Kinase Binding domain; L, Linker; RF, RING Finger; PRR, Proline-Rich Region; UBA, Ubiquitin-Associated domain. **(E)** In-cell pull-down assay mapping the HNRPA2B1-binding domain on CBL. Lysates from HEK293T cells overexpressing Flag-HNRPA2B1 were incubated with purified His-tagged full-length (WT) or truncated CBL mutants, followed by IP with an anti-His antibody. **(F)** Analysis of endogenous HNRPA2B1 ubiquitination in SH-SY5Y cells with LNK knockdown and/or CBL overexpression as indicated. **(G)** Cellular ubiquitination assay in HEK293T cells co-transfected with Flag-HNRPA2B1, shLNK, oeCBL, and HA-Ub-K27. **(H)** Cellular ubiquitination assay in HEK293T cells co-transfected with CBL, HA-Ub-K27, and either WT or K-to-R mutant Flag-HNRPA2B1 constructs to map acceptor sites. **(I–O)** SH-SY5Y cells were transfected with the indicated constructs (shNC, shLNK, empty vector, or oeCBL) and then treated with MPP^+^ (1 mM) for 24 h, unless otherwise specified. **(I)** Cell viability assessed by CCK-8 assay (n = 4). **(J)** Quantification of MDA levels (n = 3). **(K)** Quantification of lipid ROS measured by Liperfluo staining (n = 4). **(L)** Quantification of total ROS measured by DHE staining (n = 4). **(M)** Immunoblot analysis of CBL, HNRPA2B1, and GPX4 protein levels. Corresponding quantification is shown on the right (n = 3). **(N)** RT-qPCR analysis of *GPX4* mRNA levels in the indicated cell groups (n = 3). **(O)** Analysis of *GPX4* mRNA stability by RT-qPCR in the indicated SH-SY5Y cell groups treated with Act-D (5 μg/mL) for the indicated times (n = 3). Data are presented as mean ± SEM. Statistical significance was determined by one-way ANOVA with Tukey's post-hoc test (I–M) or two-way ANOVA with Tukey's post-hoc test (O). ∗P < 0.05, ∗∗P < 0.01, ∗∗∗P < 0.001. ns, not significant.

To experimentally validate this interaction, we conducted endogenous Co-IP assays in SH-SY5Y cells, which demonstrated a reciprocal interaction between CBL and HNRPA2B1 (Fig. 8A). *In vitro* glutathione S-transferase pull-down assays using purified recombinant proteins further confirmed direct binding between HNRPA2B1 and CBL (Fig. 8B). Biolayer interferometry (BLI) analysis quantified this interaction, revealing a dissociation constant (Kᴅ) of 2.2 μM (Fig. 8C).

Next, we mapped the specific domain of CBL responsible for binding to HNRPA2B1. The full-length CBL protein comprises an N-terminal tyrosine kinase binding (TKB) domain, a linker region (L), a RING finger (RF) domain that confers E3 ligase activity, a proline-rich region, and a C-terminal ubiquitin-associated (UBA) domain (Fig. 8D). We then generated a series of CBL truncation mutants. In-cell pull-down assays revealed that HNRPA2B1 specifically binds to the central region of CBL (amino acids 358–647), which encompasses the Linker and RF domains (Fig. 8E).

To functionally validate CBL as the E3 ligase responsible for HNRPA2B1 ubiquitination, we performed a series of ubiquitination assays. Analysis of endogenous ubiquitination in shLNK SH-SY5Y cells revealed that polyubiquitination of HNRPA2B1 was significantly reduced, an effect that could be rescued by overexpression of CBL (oeCBL) (Fig. 8F). Consistent with this, although LNK overexpression enhanced HNRPA2B1 polyubiquitination, this effect was abolished by CBL knockdown (shCBL), confirming CBL's essential role (Fig. S6B). To delineate specific ubiquitin linkages, ubiquitination assays were performed in HEK293T cells. Using a K27-only ubiquitin construct (HA-Ub-K27), we observed robust polyubiquitination of HNRPA2B1, confirming that CBL predominantly mediates K27-linked ubiquitination (Fig. 8G and S6C). Furthermore, site-directed mutagenesis of HNRPA2B1 identified lysine residues K100, K156, and K161 as the primary acceptor sites for CBL-mediated ubiquitination (Fig. 8H).

Finally, we performed rescue experiments to functionally link this signaling cascade to ferroptosis in an MPP^+^-induced model of neuronal injury. LNK knockdown enhanced the viability of SH-SY5Y cells exposed to MPP^+^ and protected them from ferroptosis, as evidenced by increased cell survival (Fig. 8I) and significantly reduced levels of MDA, lipid ROS, and total ROS (Fig. 8J–L). Notably, this protective effect was significantly attenuated upon CBL reintroduction into LNK knockdown cells. At the molecular level, both the elevated HNRPA2B1 protein levels and the concomitant increases in GPX4 protein and mRNA levels observed in shLNK cells were largely reversed by CBL overexpression (Fig. 8M and N). Additionally, CBL overexpression abrogated the prolonged *GPX4* mRNA half-life observed in LNK-knockdown cells (Fig. 8O).

Collectively, these findings demonstrate that LNK functions as an adaptor protein that engages the E3 ligase CBL, promoting CBL's catalytic activity toward HNRPA2B1. This LNK-dependent activation of CBL drives K27-linked ubiquitination and proteasomal degradation of HNRPA2B1, which in turn destabilizes *GPX4* mRNA and sensitizes neurons to ferroptosis.

### 3.8 LNK-dependent phosphorylation and nuclear translocation of CBL are required for HNRPA2B1 ubiquitination

**Fig. 9:**
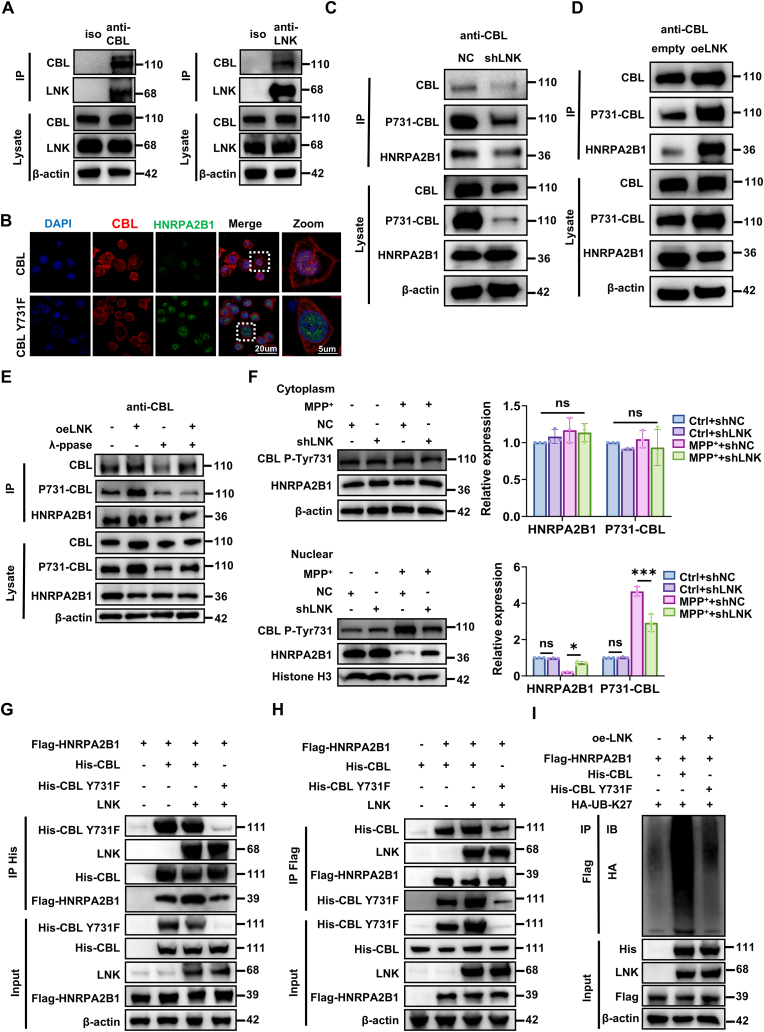
**LNK-dependent phosphorylation and nuclear translocation of CBL is required for HNRPA2B1 ubiquitination** **(A)** Endogenous co-IP assay in SH-SY5Y cells showing the interaction between LNK and CBL. (B) Representative immunofluorescence of HNRPA2B1 (green) and CBL (WT or Y731F, red) in MPP^+^-treated SH-SY5Y cells (1 mM, 24 h). Nuclei are stained with DAPI (blue). Scale bars, 20 μm. **(C)** Co-IP assay showing that LNK knockdown reduces the interaction between endogenous CBL and HNRPA2B1 in SH-SY5Y cells following MPP^+^ treatment. **(D)** Co-IP assay showing that LNK overexpression enhances the interaction between endogenous CBL and HNRPA2B1 in SH-SY5Y cells following MPP^+^ treatment. **(E)** Co-IP assay demonstrating that the CBL-HNRPA2B1 interaction is phosphorylation-dependent. Lysates from LNK-overexpressing SH-SY5Y cells following MPP^+^ treatment were treated with or without λ-phosphatase prior to IP. **(F)** Subcellular fractionation and immunoblot analysis of protein levels in cytoplasmic and nuclear fractions from shNC and shLNK cells treated with or without MPP^+^. Right: Corresponding quantification of band intensities (n = 3). **(G)** Co-IP assay in HEK293T cells. His-tagged proteins (WT CBL or Y731F mutant) were immunoprecipitated (IP: His) from lysates of cells co-transfected with the indicated constructs. IB for Flag-HNRPA2B1. **(H)** Reciprocal co-IP assay in HEK293T cells. Flag-HNRPA2B1 was immunoprecipitated (IP: Flag) from lysates of cells co-transfected with the indicated constructs. IB for His-CBL. **(I)** Ubiquitination assay. HEK293T cells were co-transfected with Flag-HNRPA2B1, HA-Ub-K27, and the indicated LNK and CBL constructs. HNRPA2B1 polyubiquitination was assessed by IP with an anti-Flag antibody followed by IB with an anti-HA antibody. Data are presented as mean ± SEM. Statistical significance was determined by two-way ANOVA with Tukey's post-hoc test (F). ∗∗P < 0.01, ∗∗∗P < 0.001; ns, not significant.

To elucidate the regulatory mechanism of the LNK–CBL–HNRPA2B1 axis, we first confirmed the interaction between LNK and its known partner [35], the E3 ligase CBL, using endogenous Co-IP (Fig. 9A). Subcellular fractionation analyses revealed that LNK is a cytosolic protein excluded from the nucleus, suggesting that it orchestrates downstream nuclear events from the cytoplasm. To delineate the structural basis of the LNK–CBL interaction, pull-down assays with CBL truncation mutants were performed. These experiments demonstrated that LNK specifically bound to the N-terminal TKB domain (residues 1–357) of CBL (Fig. S7A). Based on these findings, molecular docking simulations were conducted to model the interaction between LNK and the phosphorylated TKB domain of CBL. The predicted model indicated a stable complex stabilized by extensive hydrogen bonds and electrostatic interactions, providing a structural rationale for the direct binding of LNK to CBL (Fig. S7B).

Given that HNRPA2B1 is a nuclear protein, we investigated how the cytosolic LNK–CBL complex targets a nuclear substrate. Previous studies have reported that CBL's nuclear import and its E3 ligase activity toward nuclear substrates critically depend on phosphorylation at tyrosine 731 (Tyr731) [[37], [38], [39]]. To test this, we examined the subcellular localization of a phospho-dead CBL mutant (Y731F). Immunofluorescence analysis demonstrated that, while WT CBL exhibited both cytoplasmic and nuclear distribution, the Y731F mutant was predominantly retained in the cytoplasm. Notably, cytoplasmic sequestration of the CBL Y731F was accompanied by a significant increase in the nuclear abundance of its substrate, HNRPA2B1 (Fig. 9B). Collectively, these findings indicate that Tyr731 phosphorylation is essential for CBL nuclear translocation and the subsequent regulation of HNRPA2B1 levels.

Next, we examined the role of LNK and its phosphorylation in modulating the CBL–HNRPA2B1 interaction. Co-IP assays demonstrated that LNK knockdown significantly reduced the endogenous CBL–HNRPA2B1 interaction in SH-SY5Y cells (Fig. 9C), whereas LNK overexpression enhanced it (Fig. 9D), establishing LNK as a critical facilitator of complex formation. Treatment of cell lysates with λ-phosphatase to remove phosphate groups completely abolished the LNK-enhanced CBL–HNRPA2B1 interaction (Fig. 9E), indicating that this effect is phosphorylation-dependent.

Based on these findings, we investigated the subcellular localization of these proteins under stress conditions. Subcellular fractionation of MPP^+^-treated cells showed a significant accumulation of phosphorylated CBL (P-Tyr731-CBL) and a corresponding reduction in nuclear HNRPA2B1 in control cells. Notably, LNK knockdown abolished the stimulus-induced nuclear import of P-Tyr731-CBL, thereby stabilizing nuclear HNRPA2B1, while cytoplasmic levels of these proteins remained largely unchanged (Fig. 9F). Conversely, LNK overexpression enhanced nuclear accumulation of P-Tyr731-CBL and accelerated the degradation of nuclear HNRPA2B1 following MPP^+^ treatment (Fig. S7C). These results demonstrate that LNK acts as an upstream regulator of CBL nuclear translocation and its subsequent modulation of HNRPA2B1.

To confirm that the interaction between CBL and HNRPA2B1 strictly depends on CBL phosphorylation at Y731, we performed reciprocal Co-IP assays in HEK293T cells. Immunoprecipitation of His-tagged CBL revealed that LNK enhanced the binding of WT CBL to Flag-HNRPA2B1, whereas the CBL-Y731F mutant failed to interact (Fig. 9G). Reciprocal immunoprecipitation yielded concordant results: Flag-HNRPA2B1 co-precipitated with WT His-CBL, but not with the Y731F mutant (Fig. 9H). This loss of interaction directly translated into functional impairment, and an in-cell ubiquitination assay demonstrated that the CBL-Y731F could not mediate K27-linked polyubiquitination of HNRPA2B1, even in the presence of LNK (Fig. 9I).

Collectively, these findings delineate a precise spatiotemporal mechanism: cytosolic LNK promotes CBL phosphorylation at Tyr731, which serves as a molecular license for CBL nuclear translocation, enabling it to ubiquitinate and degrade its nuclear substrate HNRPA2B1.

### 3.9 Lifitegrast directly binds and inhibits LNK to ameliorate MPP^+^/MPTP-induced DA neurodegeneration and ferroptosis

**Fig. 10:**
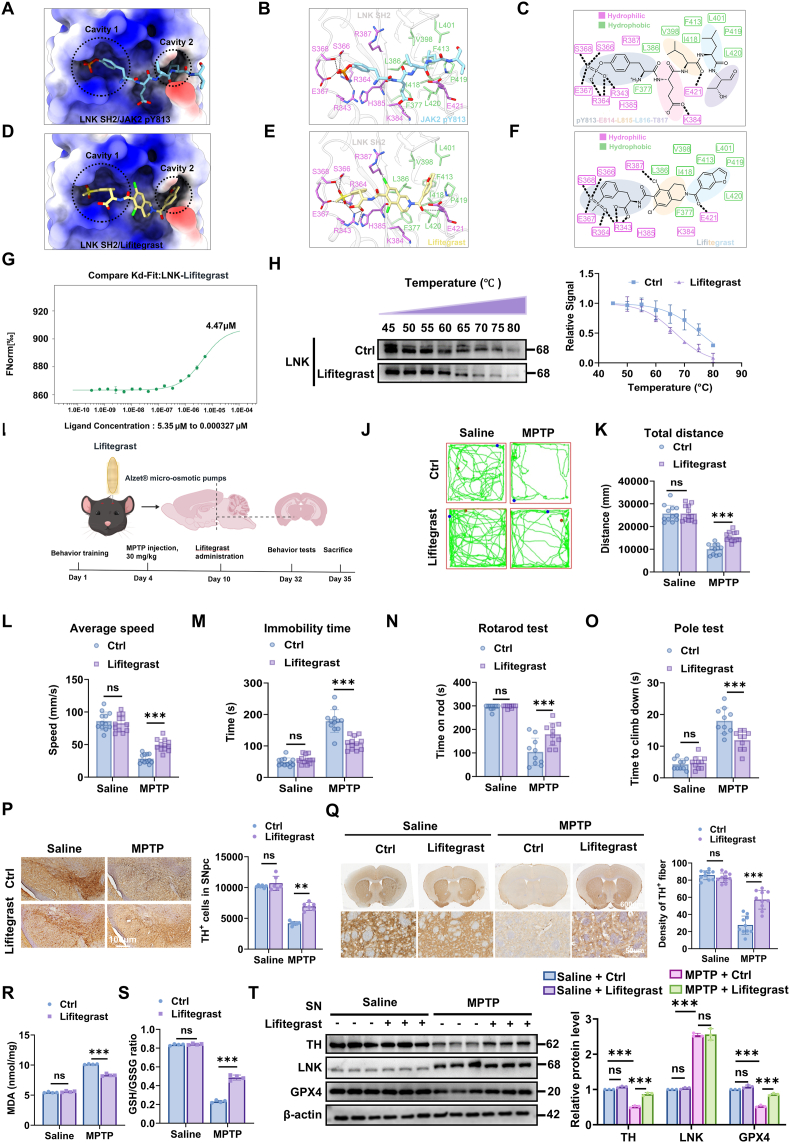
**Liftegrast ameliorates motor deficits and neurodegeneration in an MPTP-induced mouse model of PD (A**–**C)** The binding mode and detailed interactions between LNK SH2 domain and JAK2 pY813 phosphopeptide. **(D**–**F)** The binding mode and detailed interactions between LNK SH2 domain and Lifitegrast. **(G)** Microscale thermophoresis (MST) analysis of the interaction between purified LNK protein and Liftegrast, yielding a dissociation constant (Kd) of 4.476 × 10^−6^ M (n = 3). **(H)** Cellular thermal shift assay (CETSA) in SH-SY5Y cells following MPP^+^ treatment. Left: Representative immunoblots showing LNK protein levels after heating. Right: Quantification of the melting curves for LNK protein (n = 3). **(I)** Schematic of the experimental design for the MPTP mouse model and Liftegrast treatment via Alzet osmotic mini-pumps. **(J**–**O)** Behavioral tests performed on mice from different treatment groups (Saline + Ctrl, Saline + Liftegrast, MPTP + Ctrl, MPTP + Liftegrast). **(J)** Representative traces from the open-field test (n = 12). **(K)** Total distance traveled (n = 12). **(L)** Average speed (n = 12). **(M)** Immobility time in the tail suspension test (n = 12). **(N)** Time on rod in the rotarod test (n = 12). **(O)** Time to climb down in the pole test (n = 12). **(P)** Representative immunohistochemistry images showing TH-positive neurons in the SNpc. Scale bar, 100 μm. Quantification of TH-positive cell number in the SNpc is shown on the right (n = 6). **(Q)** Representative immunohistochemistry images of TH-positive fibers in the striatum. The bottom image displays a higher magnification of the region indicated in the top panel. Scale bars, 600 μm (top) and 50 μm (bottom). Quantification of relative TH-positive fiber density is shown on the right (n = 10). **(R**–**T)** Biochemical analysis of SN tissue homogenates. **(R)** MDA levels (n = 4). **(S)** GSH/GSSG ratio (n = 4). **(T)** Immunoblot analysis of TH, LNK, and GPX4 levels in SN tissues. Right: Quantification of relative protein levels (n = 3). Data are presented as mean ± SEM. Statistical analyses were performed as follows: (H) Non-linear regression with an extra sum-of-squares F-test to compare thermal shift curves; (K–T) Two-way ANOVA with Tukey's post-hoc test. ∗P < 0.05, ∗∗P < 0.01, ∗∗∗P < 0.001; ns, not significant.

The crystal structure of the LNK SH2 domain bound to the JAK2 pY813 phosphopeptide (PDB ID: 7R8W) provides a structural framework for identifying small molecules that target LNK [40]. Key residues in JAK2, pY813 and L816, are crucial for substrate recognition. Specifically, pY813 inserts into the phosphotyrosine-binding pocket (cavity 1) of the SH2 domain, forming extensive salt bridges and hydrogen bonds with R343, R354, E367, and S368, while L816 occupies a hydrophobic pocket (cavity 2) and engages in hydrophobic interactions with V398, L401, F413, I418, P419, and L420 (Fig. 10A–C and S8A). Based on the LNK SH2 domain binding interface, we conducted a virtual screening using the Drug-lib database in MTiOpenScreen, which contains 7173 clinically approved drugs. From this screen, the nine top-ranking compounds with the highest docking affinities were selected for further analysis: Bms-833923, nilotinib, Uk432097, lifitegrast, Mk-0893, radotinib, flezelastine, Pf-03758309, and etoposide phosphate. Most compounds preferentially occupied either cavity 1 (nilotinib, Uk432097, Mk-0893, radotinib, flezelastine, and Pf-03758309) or cavity 2 (etoposide phosphate) (Fig. S8B–J). Notably, lifitegrast occupied both cavities, forming extensive polar interactions in cavity 1 and hydrophobic interactions in cavity 2, closely resembling the binding mode of the JAK2 pY813 phosphopeptide. Specifically, the methylsulfonyl and carboxyl groups of lifitegrast mimic the phosphate moiety of pY813 by forming salt bridges and hydrogen bonds with surrounding polar residues, while the benzofuran group recapitulates the role of L816 by engaging in hydrophobic interactions with adjacent hydrophobic residues (Fig. 10D–F). Based on this dual-pocket binding profile, lifitegrast was identified as a promising candidate for targeting the LNK SH2 domain and was selected for further experimental validation.

To confirm direct binding and quantify affinity, we employed microscale thermophoresis (MST). Lifitegrast demonstrated dose-dependent binding to purified LNK protein, with a dissociation constant (Kd) of 4.476 × 10^−6^ M, supporting a direct interaction between lifitegrast and purified LNK protein under our assay conditions (Fig. 10G). Target engagement in cells was further validated using a cellular thermal shift assay, which revealed that lifitegrast significantly increased the thermal stability of endogenous LNK protein, evidenced by a rightward shift in its melting curve compared with vehicle-treated controls (Fig. 10H). These results indicate that lifitegrast directly binds to LNK and stabilizes it both *in vitro* and in situ.

We first evaluated the neuroprotective potential of lifitegrast in cellular models of PD. Treatment with MPP^+^ induced significant cell death in both primary neurons and SH-SY5Y neuroblastoma cells. Co-treatment with lifitegrast conferred a dose-dependent protective effect, with concentrations as low as 1 μM significantly improving cell viability in both models, indicating potent neuroprotective efficacy against MPP^+^-induced toxicity (Fig. S9A). To elucidate the underlying mechanism, we examined the effect of lifitegrast on the LNK–CBL interaction under neurotoxic stress. Co-IP assays in SH-SY5Y cells revealed that lifitegrast significantly attenuated the endogenous interaction between LNK and phosphorylated CBL (P731-CBL), particularly in the presence of MPP^+^ (Fig. S9B). Reciprocal Co-IP assays, in which P731-CBL was immunoprecipitated, yielded concordant results, confirming that lifitegrast disrupts the formation of the LNK–P731–CBL complex (Fig. S9C).

To assess whether lifitegrast disrupts the LNK–CBL E3 ligase axis, we examined its effect on HNRPA2B1 ubiquitination. In SH-SY5Y cells, LNK overexpression markedly enhanced K27-linked polyubiquitination of endogenous HNRPA2B1, which was substantially attenuated by lifitegrast treatment (Fig. S9D). This finding was corroborated in HEK293T cells co-expressing Flag-HNRPA2B1, LNK, and HA-Ub-K27, where lifitegrast similarly diminished LNK-induced HNRPA2B1 ubiquitination (Fig. S9E). These results demonstrate that lifitegrast effectively inhibits LNK-CBL-mediated ubiquitination of HNRPA2B1. Consistent with these findings, we examined the effect of lifitegrast on key pathological proteins in the MPP^+^-induced neurotoxicity model. As expected, MPP^+^ treatment reduced the levels of TH and the ferroptosis regulator GPX4—an effect further exacerbated by LNK overexpression. Lifitegrast treatment partially rescued the MPP^+^-induced reduction of TH and GPX4 in control cells and significantly mitigated the exacerbated protein loss in LNK-overexpressing cells (Fig. S9F).

To further validate that the neuroprotective effect of lifitegrast involves suppression of ferroptosis, we measured key biochemical markers of this cell death pathway. MPP^+^ treatment, particularly when combined with LNK overexpression, induced significant oxidative stress characterized by a decreased GSH/GSSG ratio, accumulation of the lipid peroxidation product MDA, and elevated levels of both lipids and total ROS. Notably, lifitegrast treatment significantly mitigated these pro-ferroptotic alterations in both control (empty vector) and LNK-overexpressing SH-SY5Y cells following MPP^+^ insult (Fig. S9G–J).

To validate these findings *in vivo*, we employed an MPTP-induced mouse model of PD, with lifitegrast administered continuously via Alzet osmotic mini-pumps (Fig. 10I) [41,42]. To assess the therapeutic efficacy of lifitegrast *in vivo*, we performed a series of behavioral tests. Notably, lifitegrast treatment significantly ameliorated MPTP-induced Parkinsonian motor deficits. Specifically, it reversed hypoactivity in the open-field test, restored motor coordination in the rotarod test, and alleviated bradykinesia in the pole test (Fig. 10J–O).

Consistent with these behavioral assessments, immunohistochemical analysis demonstrated that MPTP administration significantly reduced the number of TH-positive neurons in the SNpc and decreased TH-positive fiber density in the striatum. Lifitegrast treatment significantly attenuated both neuronal loss in the SNpc and the reduction of striatal TH-positive fibers (Fig. 10P and Q). Moreover, GPX4 expression in the SN was preserved in lifitegrast-treated mice (Fig. S9K). Biochemical analysis of SN tissue demonstrated that MPTP treatment induced pronounced oxidative stress, reflected by increased MDA levels and a decreased GSH/GSSG ratio. Lifitegrast treatment significantly ameliorated these oxidative changes (Fig. 10R and S). Western blot analysis of tissue lysates from the SN and striatum further confirmed these findings. Lifitegrast treatment prevented the MPTP-induced downregulation of TH and GPX4 proteins in both brain regions, without affecting total LNK protein abundance (Fig. 10T and S9L). Collectively, these *in vivo* data demonstrate that lifitegrast exerts potent neuroprotective effects in a MPTP-induced PD model by mitigating motor deficits, DA neuron loss, and ferroptosis-related pathology.

## 4 Discussion

1. This study defines a signaling axis centered on LNK that governs dopaminergic neuronal vulnerability under Parkinson's disease–associated stress conditions. Mechanistically, LNK interacts with the E3 ubiquitin ligase CBL and promotes its phosphorylation at Tyr731, driving CBL nuclear accumulation. Nuclear CBL subsequently triggers ubiquitination of the m^6^A-associated RNA-binding protein HNRNPA2B1, promoting its proteasomal degradation. This cascade diminishes GPX4 mRNA stability and reduces GPX4 protein levels, thereby compromising the cell's primary enzymatic defense against iron-dependent lipid peroxidation [14,25].
2. In MPP^+^/MPTP-induced PD models, LNK expression is upregulated in neurons and exacerbates PD progression by promoting ferroptosis. However, the mechanisms underlying LNK upregulation remain unclear. Previous studies have identified neuroinflammatory cytokines [43,44], oxidative stress mediators [45,46], metabolic perturbations [47,48], mitochondrial dysfunction [3,49], and α-synuclein aggregation [1,4,15,50] as factors contributing to neuronal ferroptosis in PD models. These upstream pathological signals may contribute to LNK upregulation in response to MPP^+^/MPTP challenge.
3. LNK expression was also markedly elevated in PBMCs from patients with PD and positively correlated with motor severity. However, PBMCs are sensitive to systemic factors, including pharmacological interventions and inflammatory comorbidities. Our current cohort size precluded detailed stratification by disease duration, medication exposure, or inflammatory comorbidities; future validation in larger, multicenter cohorts with covariate-adjusted models will be critical to disentangle PD-specific associations from systemic inflammatory confounders.
4. Both global and dopaminergic neuron–specific LNK ablation attenuated TH loss and motor deficits. Previous work established LNK as a negative regulator of cytokine signaling in hematopoietic systems [18,19] and a modulator of neural stem cell proliferation [20,21]. Our study extends these findings by positioning LNK as a vulnerability node that amplifies ferroptotic stress signaling in PD [51]. Although an interaction between LNK and CBL has been suggested [34,35], we identify a nuclear function for CBL in this context. Unlike CBL's canonical role in downregulating receptor tyrosine kinases [37,38], nuclear *p*-Tyr731-CBL functions as an E3 ligase for HNRNPA2B1. This axis links post-translational control to the epitranscriptomic stability of GPX4, a central suppressor of ferroptosis, by reducing the abundance of the stabilizing reader HNRNPA2B1. Although our results support HNRNPA2B1's role in maintaining the stability of m^6^A-modified GPX4 mRNA in dopaminergic neurons, we cannot rule out other major targets of HNRNPA2B1 involved in ferroptosis. Future high-throughput profiling strategies will be required to comprehensively map the HNRNPA2B1 target landscape in dopaminergic neurons [42,51,52].
5. Lifitegrast—an FDA-approved LFA-1 antagonist—is repositioned as a neuronal LNK modulator in this study [[53], [54], [55]]. Lifitegrast treatment preserved the HNRNPA2B1–GPX4 axis and attenuated neurodegeneration in our models. However, we do not establish lifitegrast as an immediately translatable PD therapeutic. Potential confounding effects from LFA-1/ICAM-1 blockade and systemic immunomodulation should be considered, and brain exposure after systemic delivery remains unknown. Accordingly, future work should measure lifitegrast levels in plasma and brain tissue under the pump dosing used in MPTP mice, together with target engagement (e.g., LNK signaling readouts).
6. Despite establishing a comprehensive mechanistic framework, this study has several limitations. First, our findings await validation in advanced human-relevant models, such as patient-derived induced pluripotent stem cell (iPSC)-derived dopaminergic neurons Second, MPTP neurotoxicity encompasses multiple cell death modalities, including apoptosis, necroptosis, and pyroptosis. Our current data demonstrate that LNK exacerbates dopaminergic loss and motor deficits primarily via the ferroptotic axis, but its modulation potential for other death pathways remains to be fully characterized. The use of pathway-specific inhibitors (e.g., Z-VAD-FMK, Necrostatin-1, VX-765) and molecular markers (e.g., cleaved Caspase-3, *p*-MLKL, GSDMD) are required to determine whether other death pathways are involved in LNK-mediated dopaminergic loss and motor deficits.
7. In conclusion, this study establishes the LNK-CBL-HNRNPA2B1-GPX4 axis as a critical determinant of ferroptotic vulnerability in PD and lifitegrast as a neuronal LNK modulator with therapeutic potential.

## Ethical compliance

All research practices conformed to the ethical guidelines established by the Committee on Publication Ethics (COPE) and the Singapore Statement on Research Integrity.

## Human studies

The study protocol involving human participants was reviewed and approved by the Institutional Review Board (IRB) of the Medical College of Yangzhou University (Approval No. YXYLL-2025-107) and was conducted in accordance with the ethical principles of the Declaration of Helsinki. Prior to inclusion in the study, all participants provided written informed consent. All patient data were anonymized to protect privacy rights.

## Animal studies

All animal procedures were approved by the Institutional Animal Care and Use Committee (IACUC) of Yangzhou University (Protocol No. 202403246), which serves as the designated local authority for animal welfare. Experiments were conducted in strict accordance with the Guide for the Care and Use of Laboratory Animals of the National Institutes of Health (NIH) and complied with the U.S. Public Health Service's Policy on Humane Care and Use of Laboratory Animals. The study is reported in accordance with the Animal Research: Reporting of In Vivo Experiments (ARRIVE) guidelines. To mitigate potential confounding effects of hormonal fluctuations associated with the estrous cycle and to maintain consistency with established models in the field, only male C57BL/6J mice were used in this study.

## Funding

This study was supported by the National Natural Science Foundation of China (81771689, 81373130, and 81001308), the Undergraduate Science and Technology Innovation Fund of Yangzhou University (Grant No. XCX20240831), and the Jiangsu Province College Students’ Innovation and Entrepreneurship Training Program (Grant No. 202411117253Y).

## CRediT authorship contribution statement

**Ziqi Liu:** Investigation, Methodology, Writing – original draft. **Ruoxun Wang:** Formal analysis, Investigation. **Min Shen:** Formal analysis, Investigation. **Xinrui Lan:** Investigation. **Weixing Yan:** Investigation, Resources. **Sainan Wang:** Validation. **Mingfeng Jiang:** Data curation. **Rongqing Li:** Visualization. **Jie Zhao:** Investigation, Software. **Qicheng Wang:** Resources. **Xinyi Xv:** Project administration. **Jingwen Zhou:** Project administration. **Xin Pan:** Conceptualization, Supervision, Writing – review & editing. **Wei Li:** Conceptualization, Funding acquisition, Supervision. **Weijuan Gong:** Conceptualization, Funding acquisition, Supervision. **Li Qian:** Conceptualization, Funding acquisition, Supervision, Writing – review & editing.

## Declaration of competing interest

The authors declare that they have no known competing financial interests or personal relationships that might be perceived to influence the work described in this article.

## Data Availability

Data will be made available on request.
