# Supplementary material for: A LNK–CBL–HNRPA2B1–GPX4 signaling axis mediates dopaminergic neuron vulnerability to ferroptosis in Parkinson's disease
Source: Redox Biol. 2026 Jan 23;90:104039. doi: 10.1016/j.redox.2026.104039 (PMC12876700; doi:10.1016/j.redox.2026.104039)
Supplement: Multimedia component 1 [file mmc1.docx]

Supporting Information 1

Supplementary Figure1-9

**A LNK–CBL–HNRPA2B1–GPX4 signaling axis mediates dopaminergic neuron vulnerability to ferroptosis in Parkinson's disease**

**
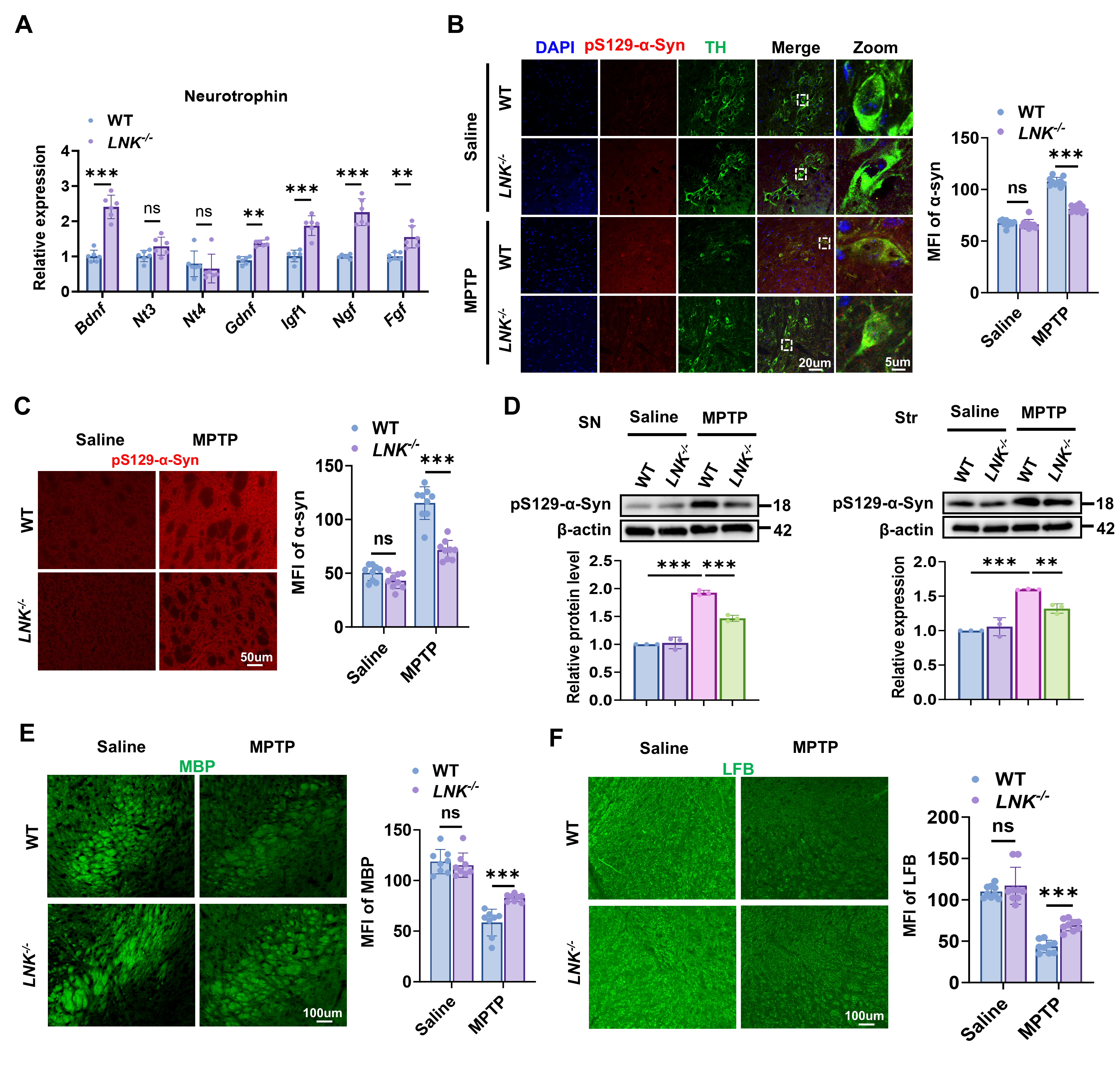
**

**Figure S1. LNK deficiency mitigates MPTP-induced alterations in neurotrophic factors, α-synuclein pathology, and demyelination (A)** Relative mRNA expression of neurotrophic factors (*Bdnf*, *Nt3*, *Nt4*, *Gdnf*, *Igf1*, *Ngf*, *Fgf*) in the SNpc of WT and *Lnk*^⁻/⁻^ mice following MPTP treatment, measured by RT-qPCR (n = 6). **(B)** Representative co-immunofluorescence images for pS129-α-Syn (red), TH (green), and DAPI (blue) in the SNpc. Right panels are magnified views of the boxed regions. Far right: Quantification of pS129-α-Syn mean fluorescence intensity (MFI) within TH-positive neurons (n = 6). Scale bars, 20 µm (main) and 5 µm (zoom). **(C)** Representative immunofluorescence images of pS129-α-Syn (red) in the striatum. Right: Quantification of pS129-α-Syn MFI (n = 6). Scale bar, 50 µm. **(D)** Representative immunoblots and quantification of pS129-α-Syn protein levels in the SN and striatum from the indicated groups (n = 3). **(E)** Representative immunofluorescence images of MBP (green) in the SNpc. Right: Quantification of MBP MFI (n = 6). Scale bar, 100 µm. **(F)** Representative LFB staining for myelin in the SNpc. Right: Quantification of LFB MFI (n = 6). Scale bar, 100 µm.Data are presented as mean ± SEM. Statistical significance was determined using a two-tailed unpaired Student’s t-test (I, J) or two-way ANOVA with Tukey’s post-hoc test (D, H). **P* < 0.01, ***P* < 0.001. ns, not significant.

**
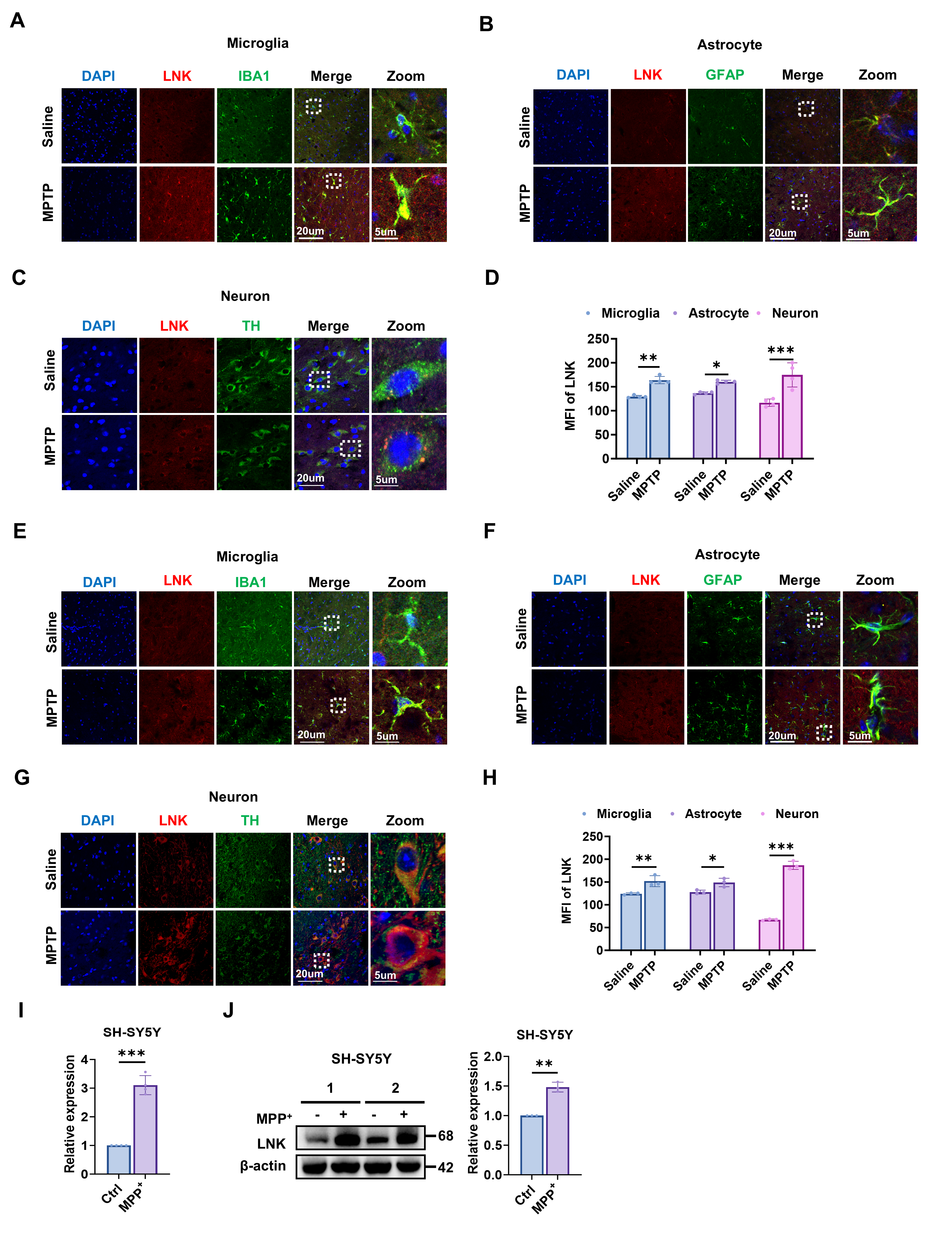
**

**Figure S2. LNK is upregulated in *in vivo* and *in vitro* models of Parkinson’s disease**

**(A-C)** Representative FISH images for *Lnk* mRNA (red) co-stained with immunofluorescence for microglia (IBA1, green, A), astrocytes (GFAP, green, B), and DA neurons (TH, green, C) in the SNpc of saline- or MPTP-treated mice. Nuclei were counterstained with DAPI (blue). Scale bars, 20 µm (main) and 5 µm (zoom). **(D)** Analysis of the *Lnk* mRNA FISH signal intensity from images as shown in (A-C). n = 4. **(E-G)** Representative co-immunofluorescence images for LNK protein (red) with markers for microglia (IBA1, green, E), astrocytes (GFAP, green, F), and DA neurons (TH, green, G) in the SNpc of saline- or MPTP-treated mice. Nuclei were counterstained with DAPI (blue). Scale bars, 20 µm (main) and 5 µm (zoom). **(H)** Quantification of LNK protein MFI from images as shown in (E-G) (n = 3). **(I)** Relative mRNA expression of *LNK* in human SH-SY5Y cells treated with vehicle (Ctrl) or MPP⁺ (500 µM, 24 h), determined by RT-qPCR (n = 4). **(J)** Representative immunoblot and quantification of LNK protein in SH-SY5Y cells treated with vehicle (Ctrl) or MPP⁺. β-actin served as a loading control (n = 3). Data are presented as mean ± SEM. Statistical significance was determined by two-way ANOVA with Tukey’s post-hoc test (D, H) or one-way ANOVA with Dunnett’s test (I, J). **P* < 0.05, ***P* < 0.01, ****P* < 0.001. ns, not significant.

**

**

**Figure S3. LNK overexpression sensitizes SH-SY5Y cells to ferroptosis** All experiments were performed in SH-SY5Y cells stably overexpressing LNK (oeLNK) or an empty vector control (empty). **(A)** Immunoblot and quantification confirming LNK overexpression efficiency (n = 3). **(B)** Quantification of cell survival (CCK8 assay) in cells treated with or without MPP⁺ (n = 4). **(C)** Quantification of the GSH/GSSG ratio (n = 3). **(D)** Quantification of MDA levels (n = 4). **(E)** Quantification of lipid ROS levels (Liperfluo), n = 4. **(F)** Quantification of general ROS levels (DHE), n = 4. **(G)** Immunoblot and quantification of GPX4 protein in cells treated with or without MPP⁺ (1 mM, 24 h), n = 3. **(H)** Immunoblot and quantification of GPX4 after co-treatment with MPP⁺ and Liproxstatin-1 (Lip-1, 1 µM, 24 h), n = 3. **(I)** Immunoblot and quantification of GPX4 after co-treatment with MPP⁺ and Ferrostatin-1 (Fer-1, 1 µM, 24 h), n = 3. **(J)** Quantification of cell survival after co-treatment with MPP⁺ and Lip-1 or Fer-1 (1 µM, 6 h), n = 4. **(K, L)** Quantification of lipid ROS levels after co-treatment with MPP⁺ and Lip-1 (K) or Fer-1 (L), n = 4. **(M, N)** Quantification of general ROS levels after co-treatment with MPP⁺ and Lip-1 (M) or Fer-1 (N), n = 4. Data are presented as mean ± SEM. Statistical significance was determined by a two-tailed Student’s t-test (A) or two-way ANOVA with Tukey’s post-hoc test (B-N). **P* < 0.05, ***P* < 0.01, ****P* < 0.001.

**
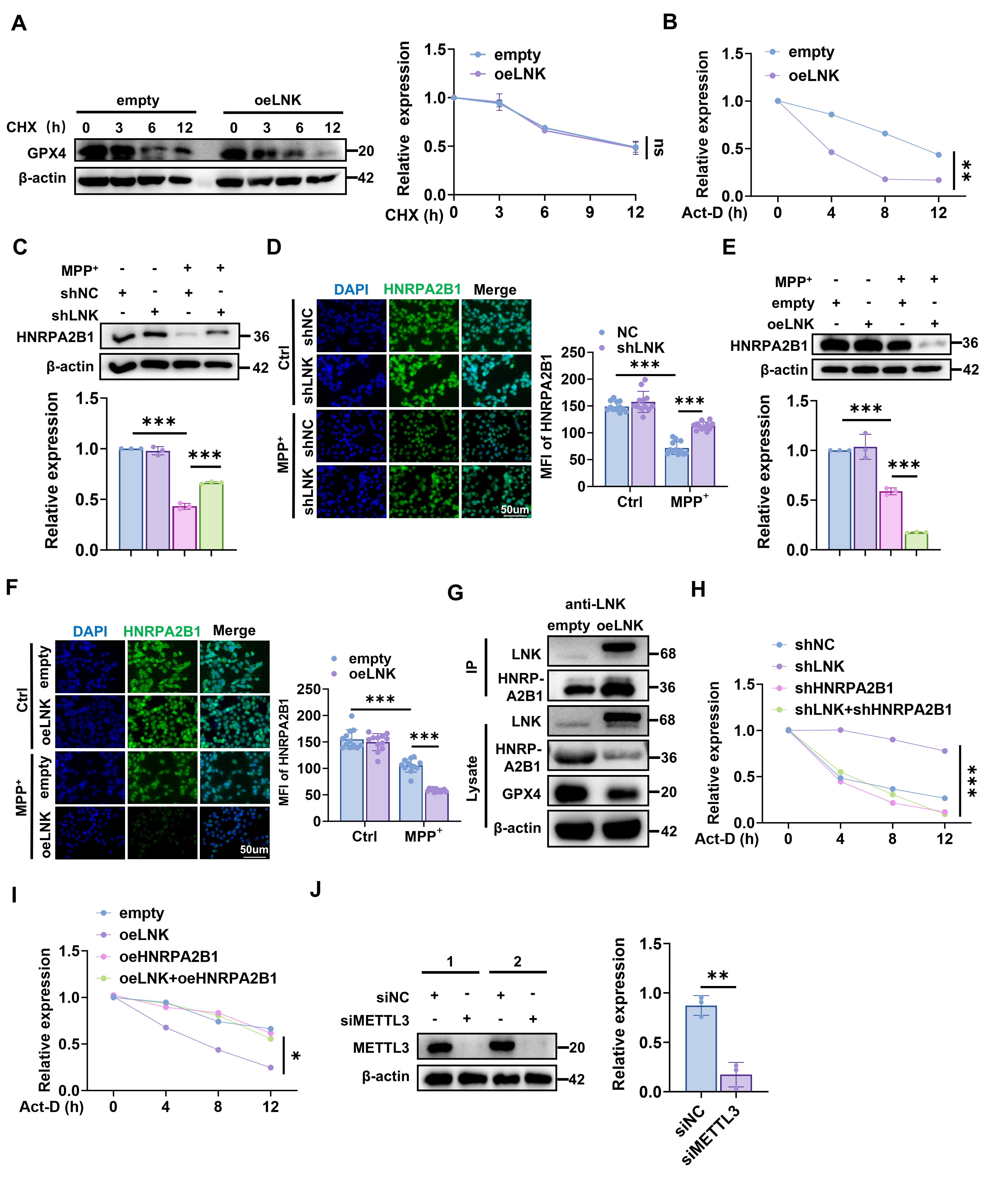
**

**Figure S4. LNK regulates *GPX4* mRNA stability in an HNRPA2B1-dependent manner** All experiments were performed in SH-SY5Y cells. **(A)** Analysis of GPX4 protein stability by immunoblot in oeLNK cells or an empty cells, treated with cycloheximide (CHX; 100 µg/mL) for the indicated times following MPP⁺ treatment (n = 3). **(B)** Analysis of *GPX4* mRNA stability by RT-qPCR in oeLNK and control cells treated with Act-D (5 µg/mL) for the indicated times following MPP⁺ treatment (n = 3). **(C)** Immunoblot analysis (top) and corresponding quantification (bottom) of HNRPA2B1 protein levels in shNC and shLNK cells, with or without MPP⁺ treatment (1 mM, 24 h), n = 3. **(D)** Representative immunofluorescence images (left) and quantification of HNRPA2B1 MFI (right) in shNC and shLNK cells, with or without MPP⁺ treatment. Nuclei were stained with DAPI (blue). Scale bar, 50 µm (n = 15). **(E)** Immunoblot analysis and quantification of HNRPA2B1 in cells transfected with an empty or oeLNK cells, with or without MPP⁺ treatment (n = 3). **(F)** Representative immunofluorescence images and MFI quantification of HNRPA2B1 in cells transfected with an empty or oeLNK cells, with or without MPP⁺ treatment. Scale bar, 50 µm (n = 15) **(G)** Co-immunoprecipitation of LNK demonstrating enhanced interaction with HNRPA2B1 in oeLNK cells compared to empty vector control with MPP⁺ treatment (n = 3). **(H)** Analysis of GPX4 mRNA stability by RT-qPCR in the indicated cells with MPP⁺ treatment (n = 3). **(I)** Analysis of *GPX4* mRNA stability by RT-qPCR in the indicated cells with MPP⁺ treatment (n = 3). **(J)** Immunoblot and corresponding quantification confirming the knockdown efficiency of METTL3 siRNA (si-METTL3) compared to a negative control siRNA (NC), n = 3. Data are presented as mean ± SEM. Statistical significance was determined by one-way ANOVA (C, E) and two-way ANOVA (A, B, D, F, H, I) or a two-tailed Student’s t-test (J). *P < 0.05, ***P < 0.001, ns, not significant.


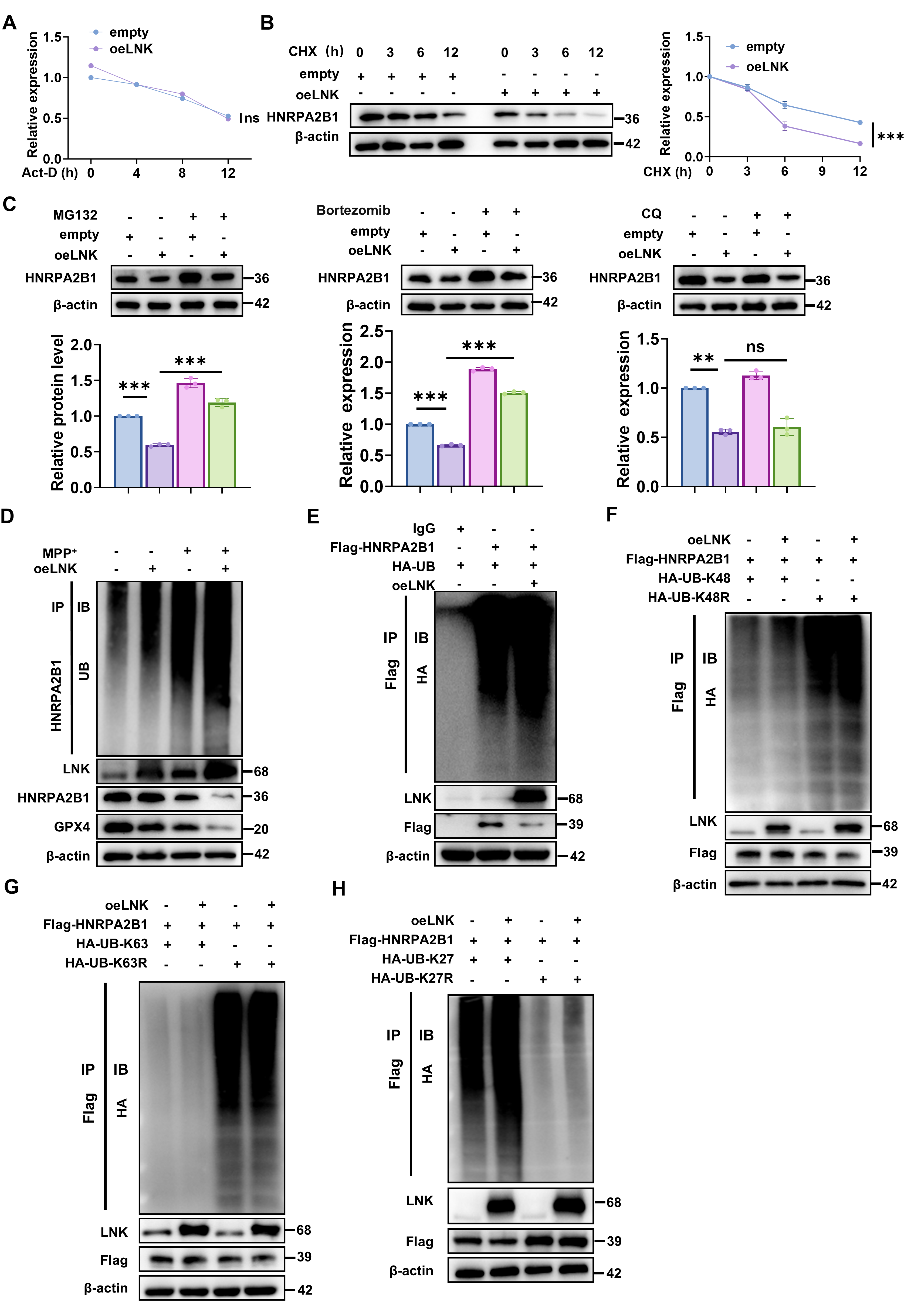


**Figure S5. LNK promotes HNRPA2B1 degradation via K27-linked ubiquitination** All experiments were performed in SH-SY5Y or HEK293T cells as specified. **(A)** RT-qPCR analysis of *HNRPA2B1* mRNA stability in oeLNK cells treated with Act-D under MPP⁺ stress (n = 3). **(B)** Immunoblot and quantification of HNRPA2B1 protein stability in oeLNK cells treated with CHX under MPP⁺ stress (n = 3). **(C)** Immunoblot of HNRPA2B1 in oeLNK cells co-treated with proteasome or lysosome inhibitors under MPP⁺ stress (n = 3). **(D)** Analysis of endogenous HNRPA2B1 ubiquitination in SH-SY5Y cells overexpressing LNK under MPP⁺ stress. **(E)** Cellular ubiquitination assay demonstrating that LNK overexpression promotes HNRPA2B1 polyubiquitination. HEK293T cells were co-transfected with Flag-HNRPA2B1, HA-Ub, and either an LNK expression vector (+) or an empty vector (-). **(F-H)** Cellular ubiquitination assays in HEK293T cells to identify the specific ubiquitin linkage type on HNRPA2B1 promoted by LNK. The assays involved co-transfection of Flag-HNRPA2B1 and oeLNK with pairs of linkage-specific HA-ubiquitin (HA-Ub) constructs. Specifically, polyubiquitination was compared using constructs that permit only K48 linkage (HA-Ub-K48) versus those in which K48 is mutated to arginine (HA-Ub-K48R) **(F)**. Parallel experiments were performed to assess K63 linkage (HA-Ub-K63 vs. HA-Ub-K63R) **(G)** and K27 linkage (HA-Ub-K27 vs. HA-Ub-K27R) **(H)**. Data are presented as mean ± SEM. Statistical significance determined by two-way ANOVA (A, B) or one-way ANOVA with Tukey’s post-hoc test (C). ***P < 0.001. ns, not significant.

**
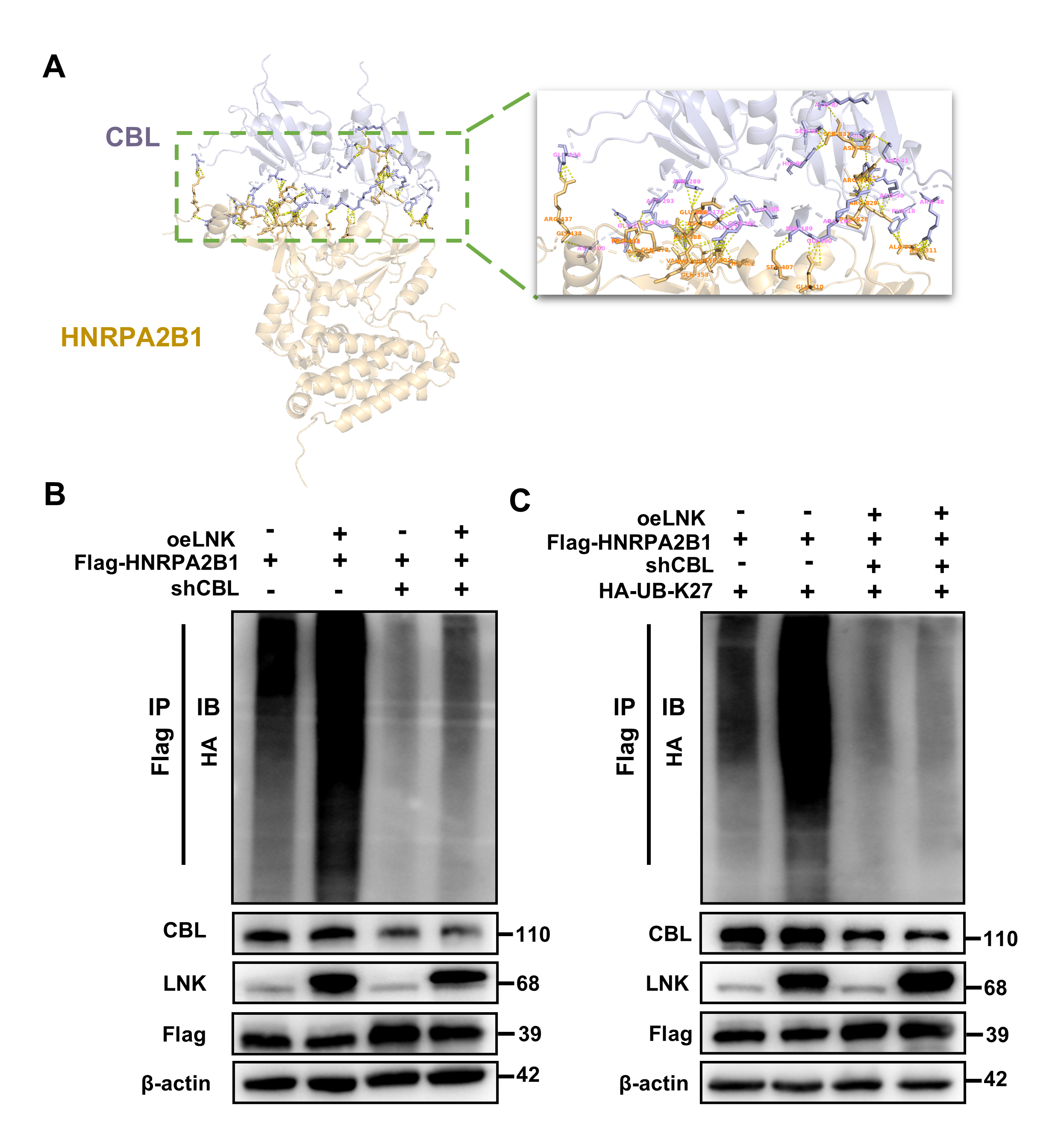
**

**Figure S6. LNK facilitates CBL-HNRPA2B1 complex formation and ubiquitination** **(A)** Molecular docking model of the CBL-HNRPA2B1 complex. The structure was predicted using AlphaFold3 and docked via the HDOCK server. The inset shows a detailed view of the binding interface, highlighting key hydrogen bonds (yellow dashes) and hydrophobic interactions. **(B)** HNRPA2B1 ubiquitination in HEK293T cells co-transfected with oeLNK and/or shCBL as indicated. **(C)** LNK promotes CBL-dependent, K27-linked ubiquitination of HNRPA2B1. HEK293T cells were co-transfected with Flag-HNRPA2B1, HA-tagged K27-only ubiquitin (HA-Ub-K27), and the indicated constructs (oeLNK or shCBL). Ubiquitinated HNRPA2B1 was detected by immunoprecipitation (IP) with an anti-Flag antibody, followed by immunoblotting (IB) for the HA tag.

**
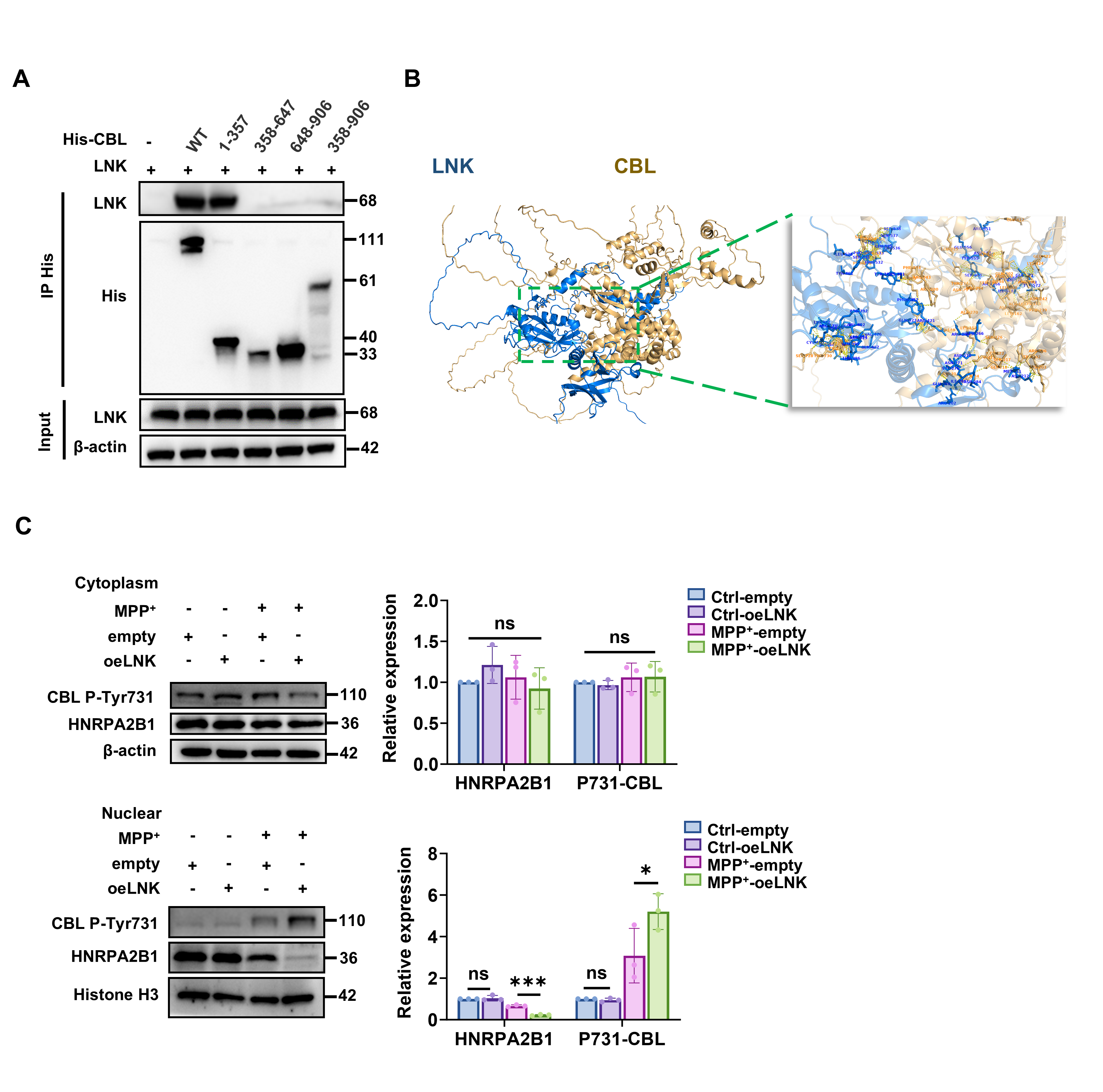
**

**Figure S7. LNK regulates the CBL-HNRPA2B1 interaction and spatiotemporal dynamics** (A) Identification of the CBL domain required for interaction with LNK. A Co-IP assay was performed using lysates from HEK293T cells expressing a series of His-tagged CBL truncations. **(B)** Molecular docking model of the LNK-CBL TKB domain complex. The simulation was performed with the CBL TKB domain (residues 1-357) in its phosphorylated state. The inset highlights key hydrogen bonds and electrostatic interactions at the binding interface. **(C)** Subcellular fractionation and immunoblot analysis of protein levels in cytoplasmic and nuclear fractions from empty and oeLNK SH-SY5Y cells treated with or without MPP⁺ (1 mM, 24 h). Right: Corresponding quantification of band intensities (n = 3). Data are presented as mean ± SEM. Statistical significance was determined by two-way ANOVA with Tukey’s post-hoc test (C). ***P < 0.001; ns, not significant.

**
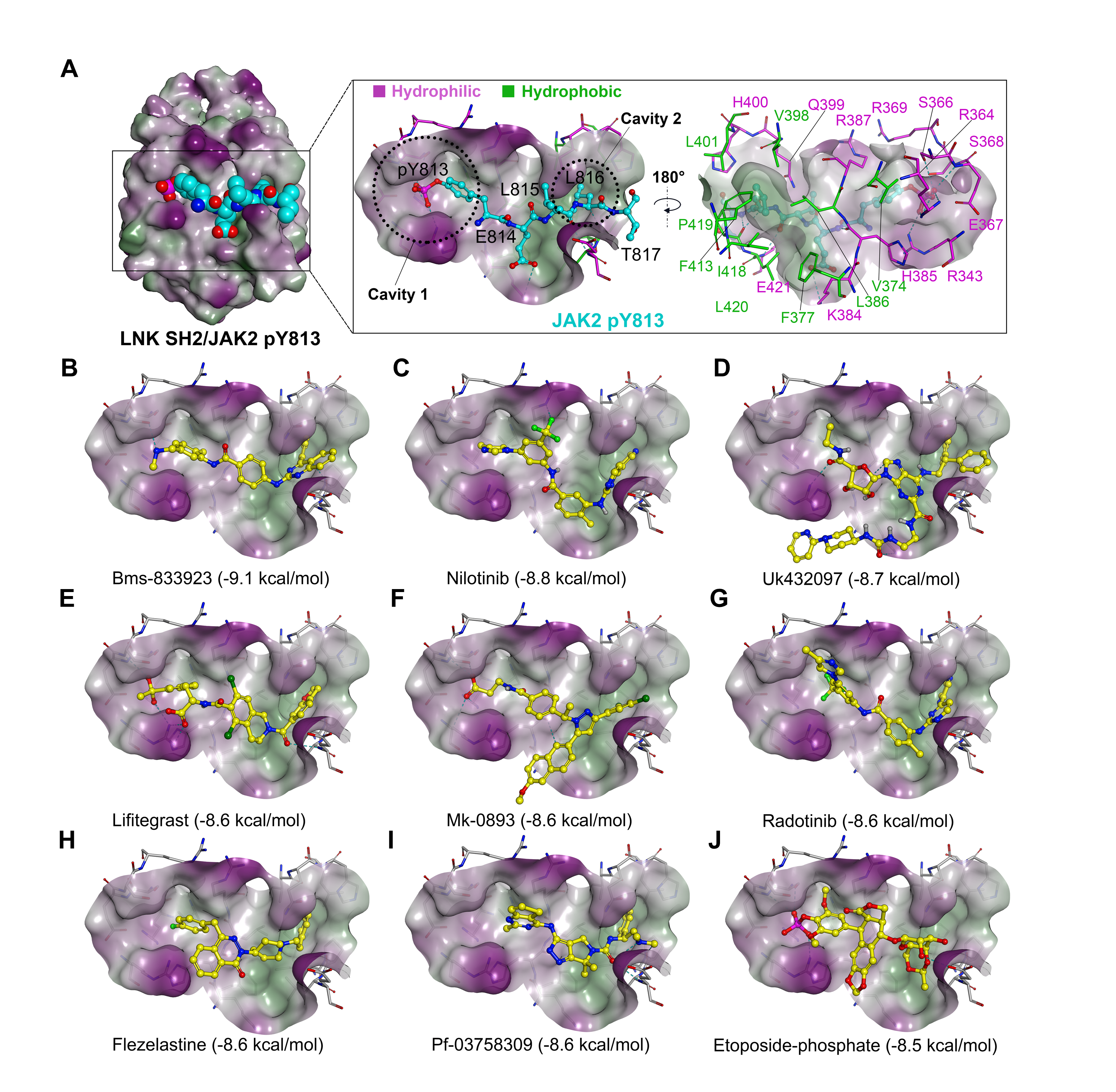
**

**Figure S8. Structure-Based Virtual Screening Identifies Potential Ligands Targeting the LNK SH2 Domain (A)** Hydrophilic and hydrophobic property analysis of the LNK SH2 domain. **(B-J)** Results of the virtual screening targeting the LNK SH2 domain. The top 9 compounds with the highest docking affinities include Bms-833923 **(B)**, Nilotinib **(C)**, Uk432097 **(D)**, Lifitegrast **(E)**, Mk-0893 **(F)**, Radotinib **(G)**, Flezelastine **(H)**, Pf-03758309 **(I)**, and Etoposide-phosphate **(J)**.

**
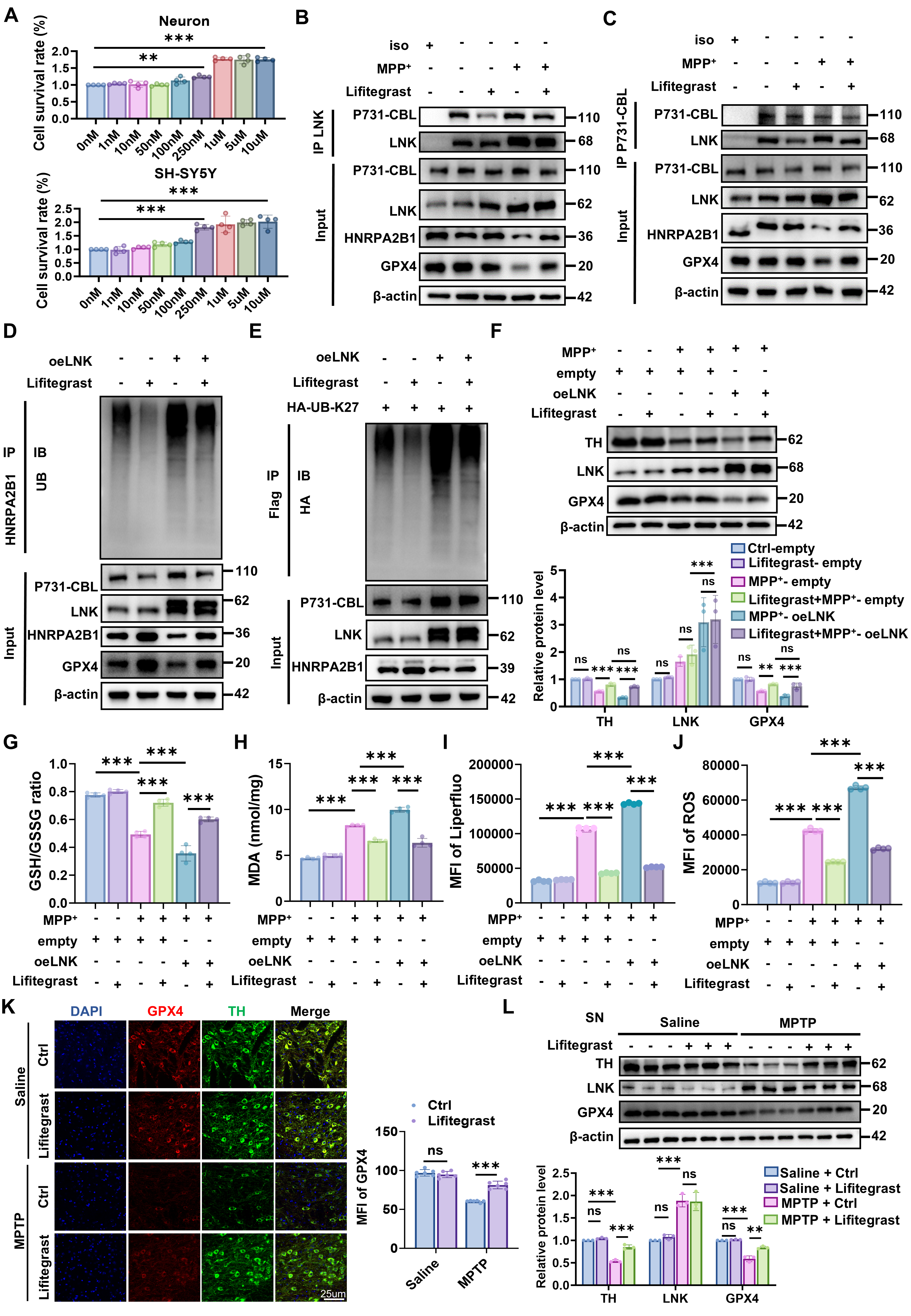
**

**Figure S9. Liftegrast protects against MPP⁺-induced neurotoxicity by inhibiting the LNK-CBL axis and ferroptosis** **(A)** Cell viability assays. Primary neurons and SH-SY5Y cells were challenged with MPP⁺ (1 mM) in the presence of increasing concentrations of Liftegrast for 24 h (n = 4). **(B)** Co-IP of endogenous LNK from SH-SY5Y cells treated with MPP⁺ (1 mM, 24 h) with or without Liftegrast (10 μM). Immunoblotting (IB) for P731-CBL and LNK. **(C)** Reciprocal co-IP of endogenous P731-CBL from SH-SY5Y cells treated as in (B). IB for LNK and P731-CBL. **(D)** Ubiquitination assay of endogenous HNRPA2B1 in SH-SY5Y cells treated with MPP⁺ in the presence or absence of Liftegrast. **(E)** Cellular ubiquitination assay. HEK293T cells were co-transfected with Flag-HNRPA2B1, HA-Ub-K27, and oeLNK or empty vector, followed by treatment with Liftegrast. K27-linked ubiquitination of HNRPA2B1 was assessed by immunoprecipitation with an anti-Flag antibody and subsequent immunoblotting with an anti-HA antibody. **(F)** Immunoblot analysis of TH, LNK, and GPX4 levels in SH-SY5Y cells transfected with empty vector or oeLNK, and treated with or without MPP⁺ and Liftegrast. Right: Quantification of protein levels (n = 3). **(G-J)** Quantification of ferroptosis markers in SH-SY5Y cells treated as in (F). (G) GSH/GSSG ratio (n = 4), (H) MDA levels (n = 4), (I) Lipid ROS levels (MFI of Liperfluo) (n = 4), and (J) total ROS levels (MFI of DCFH-DA) (n = 4). **(K)** Representative immunofluorescence images of TH (green) and GPX4 (red) in the SN of mice from different treatment groups. Nuclei are stained with DAPI (blue). Scale bar, 20 μm. Right: Quantification of the MFI of GPX4 (n = 6). **(L)** Immunoblot analysis of TH, LNK, and GPX4 levels in striatum tissues from the four groups of mice. Right: Quantification of protein levels (n = 4). Data are presented as mean ± SEM. Statistical significance was determined by one-way ANOVA (A, G-J) or two-way ANOVA (F, K, L) with Tukey’s post-hoc test. *P < 0.05, **P < 0.01, ***P < 0.001; ns, not significant.

















**Supporting Information 3**

Supplementary Table 1-3

**A LNK–CBL–HNRPA2B1–GPX4 signaling axis mediates dopaminergic neuron vulnerability to ferroptosis in Parkinson's disease**

Supplementary Table 1-1 Primer sequences for mouse tail identification

| Primer name |  |  |
| --- | --- | --- |
| WTF | 5’GTCCGACTCTCTGGCTATGTGGTA-3’ |  |
| KOF  CommonR | 5'-CGCATCGCCTTCTATCGCCT-3'  5'-GAAGAGGAGTCCATGTCATAGTCC-3' |  |
| Primer name | Upstream sequence | Downstream sequence |
| LNKLoxP-1 | CCTTAACAAATGTGGTCGCATACC | CAACCCCAACCAGCAGATAAAG |
| LNKLoxP-2 | GTAGAGTAACAGTGATGTGAGCCC | TATAAGACCTTTGCCACCCTAGC |
| DRA CRE-1 | TGGCTGTTGGTGTAAAGTGG | CCAAAAGACGGCAATATGGT |
| DRA CRE-2 | TGGCTGTTGGTGTAAAGTGG | GGACAGGGACATGGTTGACT |

Configure the reaction system：

Supplementary Table 1-2 PCR Sample Loading System

| Component | Volume |
| --- | --- |
| Forward Primer (10 μM) | 1 μL |
| Reverse Primer (10 μM)  DNA | 1 μL  1.5 μL |
| 2×Taq PCR Master Mix | 12.5 μL |
| ddH2O | 9 μL |

Amplification procedure：

Supplementary Table 1-3 PCR Program

| Stage | temperature | time | cycle number |
| --- | --- | --- | --- |
| Stage 1 | 95℃ | 5 min | 1 |
| Stage 2 | 95℃ | 30 s | 35 |
|  | 60℃ | 30 s |  |
|  | 72℃ | 45 s |  |
| Stage 3 | Default Settings of the instrument | | 1 |

The PCR amplification products were analyzed by agarose gel electrophoresis.

Supplementary Table 2 The main antibodies/reagents used in the experiment

| Antibody name | Manufacturer | Product Number | Dilution ratio（WB） | Dilution ratio（IF/IP） | Dilution ratio (IP/RIP/MERIP) |
| --- | --- | --- | --- | --- | --- |
| TH | Servicebio | GB11181-100 | 1：500 | 1:200 |  |
| TH | Servicebio | GB12181-100 | 1:500 | 1:200 |  |
| LNK | Santa | sc-514025 | 1:500 | 1:200 | 1：20 |
| α-Syn | CST | 45083SF | 1:1000 | 1:200 |  |
| MBP | Santa | sc-271524 |  | 1.200 |  |
| FluoroMyelin™ Green | Thermo Fisher | F34651 | 1:1000 |  |  |
| β-Actin | CST | 3700S | 1:1000 |  |  |
| GPX4 | HUABIO | AB_3070665 | 1:10000 | 1:100 |  |
| GPX4 | ABclonal | A13309 | 1:1000 | 1:100 |  |
| ALOX12 | ABclonal | A14703 | 1:1000 |  |  |
| ACSL3 | ABclonal | A11679 | 1:1000 |  |  |
| GFAP | abcam | ab7260 | 1:1000 |  |  |
| IBA1 | WAKO | 019-19741 | 1:1000 |  |  |
| HNRPA2B1 | Santa | sc-32316 | 1:500 | 1:200 | 1:20 |
| HNRPA2B1 | ABclonal | A27189 | 1:2000 | 1:500 | 1:20 |
| METTL3 | ABclonal | A19079 | 1:2000 |  | 1:20 |
| Flag-tag | ABclonal | AE092 | 1:10000 |  | 1:20 |
| His-tag | ABclonal | AE086 | 1:10000 |  | 1:20 |
| GST-Tag | ABclonal | AE077 | 1:10000 |  | 1:20 |
| ubiquitin | proteintech | 10201-2-AP | 1:5000 |  | 1:20 |
| CBL | ABclonal | A0732 | 1:2000 | 1:500 | 1:20 |
| Phospho-CBL (Tyr731) | Abmart | TA8006M | 1:2000 | 1:500 | 1:20 |
| HRP-conjugated Goat Anti-Rabbit IgG(H+L) | proteintech | SA00001-2 | 1:10000 |  |  |
| HRP-conjugated Goat Anti-Mouse IgG(H+L) | proteintech | SA00001-1 | 1:10000 |  |  |
| Multi-rAb™ Polymer HRP-Goat Anti-Rabbit | proteintech | RGAR011 | 1:10000 |  |  |
| Multi-rAb™ CoraLite® Plus 488-Goat Anti-Rabbit | proteintech | RGAR002 |  | 1:200 |  |
| Multi-rAb™ CoraLite® Plus 488-Goat Anti-Mouse | proteintech | RGAM002 |  | 1:200 |  |
| Multi-rAb™ CoraLite® Plus 594-Goat Anti-Rabbit | proteintech | RGAR004 |  | 1:200 |  |
| Multi-rAb™ CoraLite® Plus 594-Goat Anti-Mouse | proteintech | RGAM004 |  | 1:200 |  |
| Multi-rAb™ CoraLite® Plus 647-Goat Anti-Rabbit | proteintech | RGAR005 |  | 1:200 |  |
| Multi-rAb™ CoraLite® Plus 647-Goat Anti- Mouse | proteintech | RGAM005 |  | 1:200 |  |

Supplementary Table 3-1 The primer sequence used in the research project is as follows

| Primer name | Upstream sequence | | Downstream sequence |
| --- | --- | --- | --- |
| mActin | GTCATCACTATCGGCAATG | | GTGTTGGCATAGAGGTCT |
| hActin | GCGTGACATTAAGGAGAAG | | GAAGGAAGGCTGGAAGAG |
| GDNF | GGCAGTGCTTCCTAGAAGAGA | | AAGACACAACCCCGGTTTTTG |
| hLNK | GGAGCTTCACCCTCGAAATGG | | TTGAGATGCCTGACAACCTTTAC |
| mLNK | CAATACGACCTCCTTGAGCG | | TGCCCTTGAACACAGACTTG |
| IGF | GCTCTTCAGTTCGTGTGTGGA | | GCCTCCTTAGATCACAGCTCC |
| NGF | GGCAGACCCGCAACATTACT | | CACCACCGACCTCGAAGTC |
| FGF | CAGGCGGAGGCAGCTATAC | | CCTGGTTCCCTGGATAGTACC |
| BDNF | TCATACTTCGGTTGCATGAAGG | | AGACCTCTCGAACCTGCCC |
| NT3 | AGTTTGCCGGAAGACTCTCTC | | GGGTGCTCTGGTAATTTTCCTTA |
| NT4 | | TGAGCTGGCAGTATGCGAC | CAGCGCGTCTCGAAGAAGT |
| ACSL3 | | AACCACGTATCTTCAACACCATC | AGTCCGGTTTGGAACTGACAG |
| ACSL1 | | GCAACCGGGTCAAGTTGGT | CAAGTCGTTGGAGTAGTTGGG |
| ACSL4 | | ATATTCGTCACCACTCACA | AACCTTGCTCATAACATTCTT |
| GPX4 | | CGATACGCTGAGTGTGGTTT | CGGCGAACTCTTTGATCTCTT |
| GPX4 site1 | | CAAGTGGAACTTCACCAAG | CACACACTTGTGGAGCT |
| GPX4 site2 | | TGCGCGCTCCATGCACGAGTTT | CACGTTGGTGACGATGCACACGAA |
| GPX4 site3 | | CTGCCTGCAAACCTGCTGGT | CTGTTTATTCCCACAAGGTAG |
| FABP5 | | AAAGAGCTAGGAGTAGGACTGG | TGTTGCCATCACACGTAATGA |
| CYPLA2 | | AGTACATCTCCTTAGCCCCAG | GGGTCCGGGTGGATTCTTC |
| CYP2J6 | | TTAGCCACGATCTGGGCAG | CTGGGGGATAGTTCTTGGGG |
| CYP2E1 | | CGTTGCCTTGCTTGTCTGGA | AAGAAAGGAATTGGGAAAGGTCC |
| CYP2C | | ATCTGGTCGTGTTCCTAGCG | CAGTAGGCTTTGAGCCCAAATA |
| ELOVL2 | | CACGTACCTGCTCTCGATATGG | TGTGATTGCGAGGTTATACAAGG |
| ELOVL3 | | CGGATGACGCCGTAGTCAG | GGACGCTTACGCAGGATGAT |
| ELOVL5 | | ATGGAACATTTCGATGCGTCA | GTCCCAGCCATACAATGAGTAAG |
| PLA2G4 | | CAGCACATTATAGTGGAACACCA | AGTGTCCAGCATATCGCCAAA |
| APOE | | CTGACAGGATGCCTAGCCG | CGCAGGTAATCCCAGAAGC |
| COX1 | | ATGAGTCGAAGGAGTCTCTCG | GCACGGATAGTAACAACAGGGA |
| Fads2 | | TCATCGGACACTATTCGGGAG | GGGCCAGCTCACCAATCAG |
| Alox12 | | ACCTCAGACAATAGCAGCGGA | TCAACGTCCATTCAAAGTCCAG |
| Alox15 | | GGCTCCAACAACGAGGTCTAC | CCCAAGGTATTCTGACACATCC |
| LRG1 | | TTGGCAGCATCAAGGAAGC | CAGATGGACAGTGTCGGCA |
| ANXA2 | | ATGTCTACTGTCCACGAAATCCT | TGACTGACCCGTAGGCACTT |
| SREBP1 | | TGACCCGGCTATTCCGTGA | CTGGGCTGAGCAATACAGTTC |

Supplementary Table 3-2 METTL3 SIRNA

| Gene name | sense（5'-3'） | antisense（5'-3'） |
| --- | --- | --- |
| METTL3 | GCCAAGGAACAAUCCAUUGUUTT | AACAAUGGAUUGUUCCUUGGCTT |
| METTL3 | CGUCAGUAUCUUGGGCAAGUUTT | AACUUGCCCAAGAUACUGACGTT |

Supplementary Table 4 Abbreviations List

| Abbreviation | Full Term |
| --- | --- |
| Act-D | Actinomycin D |
| ANOVA | Analysis of Variance |
| BLI | Biolayer Interferometry |
| CBL | Casitas B-lineage Lymphoma |
| Co-IP | Co-Immunoprecipitation |
| Cre | Cre recombinase |
| DAPI | 4′,6-diamidino-2-phenylindole |
| DAT | Dopamine Transporter |
| Fer-1 | Ferrostatin-1 |
| FIN56 | Ferroptosis-inducing agent 56 |
| GO | Gene Ontology |
| GPX4 | Glutathione Peroxidase 4 |
| GST | Glutathione S-transferase |
| HA | Hemagglutinin |
| HEK293T | Human Embryonic Kidney 293T cells |
| HNRPA2B1 | Heterogeneous Nuclear Ribonucleoprotein A2/B1 |
| IgG | Immunoglobulin G |
| IHC | Immunohistochemistry |
| IP | Immunoprecipitation |
| Kᴅ | Dissociation Constant |
| KEGG | Kyoto Encyclopedia of Genes and Genomes |
| KO | Knockout |
| Lip-1 | Liproxstatin-1 |
| LNK | Lymphocyte-specific adapter protein (also known as SH2B3) |
| m⁶A | N⁶-methyladenosine |
| MDA | Malondialdehyde |
| MeRIP | Methylated RNA Immunoprecipitation |
| MPP⁺ | 1-methyl-4-phenylpyridinium |
| MPTP | 1-methyl-4-phenyl-1,2,3,6-tetrahydropyridine |
| NC | Negative Control |
| ns | not significant |
| oe | overexpression |
| PD | Parkinson’s Disease |
| RIP | RNA Immunoprecipitation |
| ROS | Reactive Oxygen Species |
| RSL3 | Ras-selective lethal 3 |
| RT-qPCR | Reverse Transcription-quantitative Polymerase Chain Reaction |
| SD | Standard Deviation |
| SEM | Standard Error of the Mean |
| shRNA | short-hairpin RNA |
| SNpc | Substantia nigra pars compacta |
| Str | Striatum |
| TEM | Transmission Electron Microscopy |
| TH | Tyrosine Hydroxylase |
| UB | Ubiquitin |
| WT | Wild-Type |
| MST | MicroScale Thermophoresis |
| CESTA | Cellular Thermal Shift Assay |
| BLI | Biolayer interferometry |

**Supporting Information 4**

Supplementary - Human blood sample information

**A LNK–CBL–HNRPA2B1–GPX4 signaling axis mediates dopaminergic neuron vulnerability to ferroptosis in Parkinson's disease**

Peripheral blood specimens were collected from healthy controls and patients with confirmed Parkinson's disease (PD) at Subei People's Hospital of Jiangsu Province, Yangzhou, China, between March 2025 and August 2025. Demographic data and clinical characteristics of all participants were extracted from their electronic medical records (EMRs).

**HD**

| Sample | Gender: 1 male and 2 females | Age |
| --- | --- | --- |
| 1 | 2 | 39 |
| 2 | 2 | 87 |
| 3 | 2 | 53 |
| 4 | 1 | 38 |
| 5 | 1 | 38 |
| 6 | 1 | 72 |
| 7 | 1 | 59 |
| 8 | 2 | 50 |
| 9 | 2 | 75 |
| 10 | 2 | 70 |
| 11 | 2 | 56 |
| 12 | 1 | 64 |
| 13 | 2 | 62 |
| 14 | 1 | 48 |
| 15 | 1 | 76 |
| 16 | 2 | 71 |
| 17 | 1 | 42 |
| 18 | 2 | 87 |
| 19 | 1 | 66 |
| 20 | 2 | 63 |
| 21 | 2 | 56 |
| 22 | 2 | 57 |
| 23 | 1 | 57 |
| 24 | 2 | 42 |
| 25 | 1 | 57 |
| 26 | 2 | 56 |
| 27 | 1 | 56 |
| 28 | 1 | 25 |
| 29 | 1 | 56 |
| 30 | 1 | 78 |

**PD**

| Sample | Course of the disease (years) | Gender: 1 male and 2 females | Age | UPDRS-Ⅲ |
| --- | --- | --- | --- | --- |
| 1 | 5 | 1 | 63 | 33 |
| 2 | 11 | 1 | 77 | 18 |
| 3 | 4.5 | 2 | 68 | 19 |
| 4 | 0.5 | 2 | 76 | 13 |
| 5 | 2 | 1 | 66 | 19 |
| 6 | 7 | 2 | 65 | 12 |
| 7 | 1 | 2 | 76 | 21 |
| 8 | 11 | 1 | 72 | 20 |
| 9 | 4 | 1 | 62 | 20 |
| 10 | 1.5 | 1 | 70 | 32 |
| 11 | 2 | 2 | 71 | 16 |
| 12 | 5 | 2 | 63 | 28 |
| 13 | 9 | 1 | 70 | 23 |
| 14 | 1 | 1 | 60 | 2 |
| 15 | 6 | 1 | 60 | 6 |
| 16 | 3 | 1 | 65 | 6 |
| 17 | 4 | 2 | 67 | 29 |
| 18 | 1 | 1 | 67 | 16 |
| 19 | 2 | 2 | 63 | 15 |
| 20 | 1.5 | 2 | 52 | 26 |
| 21 | 1 | 1 | 78 | 8 |
| 22 | 10 | 2 | 77 | 28 |
| 23 | 3 | 1 | 60 | 12 |
| 24 | 1 | 1 | 61 | 8 |
| 25 | 2 | 1 | 53 | 13 |
| 26 | 2 | 2 | 62 | 13 |
| 27 | 1 | 1 | 75 | 10 |
| 28 | 6 | 2 | 74 | 12 |
| 29 | 10 | 1 | 80 | 26 |
| 30 | 11 | 1 | 82 | 29 |
