# Supplementary material for: A LNK–CBL–HNRPA2B1–GPX4 signaling axis mediates dopaminergic neuron vulnerability to ferroptosis in Parkinson's disease
Source: Redox Biol. 2026 Jan 23;90:104039. doi: 10.1016/j.redox.2026.104039 (PMC12876700; doi:10.1016/j.redox.2026.104039)
Supplement: Multimedia component 2 [file mmc2.docx]

**Supplementary Table 1**

**A LNK–CBL–HNRPA2B1–GPX4 signaling axis mediates dopaminergic neuron vulnerability to ferroptosis in Parkinson's disease**

Supplementary Table 1-1 Primer sequences for mouse tail identification

| Primer name |  |  |
| --- | --- | --- |
| WTF | 5’GTCCGACTCTCTGGCTATGTGGTA-3’ |  |
| KOF  CommonR | 5'-CGCATCGCCTTCTATCGCCT-3'  5'-GAAGAGGAGTCCATGTCATAGTCC-3' |  |
| Primer name | Upstream sequence | Downstream sequence |
| LNKLoxP-1 | CCTTAACAAATGTGGTCGCATACC | CAACCCCAACCAGCAGATAAAG |
| LNKLoxP-2 | GTAGAGTAACAGTGATGTGAGCCC | TATAAGACCTTTGCCACCCTAGC |
| DRA CRE-1 | TGGCTGTTGGTGTAAAGTGG | CCAAAAGACGGCAATATGGT |
| DRA CRE-2 | TGGCTGTTGGTGTAAAGTGG | GGACAGGGACATGGTTGACT |

Configure the reaction system：

Supplementary Table 1-2 PCR Sample Loading System

| Component | Volume |
| --- | --- |
| Forward Primer (10 μM) | 1 μL |
| Reverse Primer (10 μM)  DNA | 1 μL  1.5 μL |
| 2×Taq PCR Master Mix | 12.5 μL |
| ddH2O | 9 μL |

Amplification procedure：

Supplementary Table 1-3 PCR Program

| Stage | temperature | time | cycle number |
| --- | --- | --- | --- |
| Stage 1 | 95℃ | 5 min | 1 |
| Stage 2 | 95℃ | 30 s | 35 |
|  | 60℃ | 30 s |  |
|  | 72℃ | 45 s |  |
| Stage 3 | Default Settings of the instrument | | 1 |

The PCR amplification products were analyzed by agarose gel electrophoresis.
