# Supplementary material for: A LNK–CBL–HNRPA2B1–GPX4 signaling axis mediates dopaminergic neuron vulnerability to ferroptosis in Parkinson's disease
Source: Redox Biol. 2026 Jan 23;90:104039. doi: 10.1016/j.redox.2026.104039 (PMC12876700; doi:10.1016/j.redox.2026.104039)
Supplement: Multimedia component 3 [file mmc3.docx]

**Supplementary Table 3**

**A LNK–CBL–HNRPA2B1–GPX4 signaling axis mediates dopaminergic neuron vulnerability to ferroptosis in Parkinson's disease**

Supplementary Table 3-1 The primer sequence used in the research project is as follows

| Primer name | Upstream sequence | | Downstream sequence |
| --- | --- | --- | --- |
| mActin | GTCATCACTATCGGCAATG | | GTGTTGGCATAGAGGTCT |
| hActin | GCGTGACATTAAGGAGAAG | | GAAGGAAGGCTGGAAGAG |
| GDNF | GGCAGTGCTTCCTAGAAGAGA | | AAGACACAACCCCGGTTTTTG |
| hLNK | GGAGCTTCACCCTCGAAATGG | | TTGAGATGCCTGACAACCTTTAC |
| mLNK | CAATACGACCTCCTTGAGCG | | TGCCCTTGAACACAGACTTG |
| IGF | GCTCTTCAGTTCGTGTGTGGA | | GCCTCCTTAGATCACAGCTCC |
| NGF | GGCAGACCCGCAACATTACT | | CACCACCGACCTCGAAGTC |
| FGF | CAGGCGGAGGCAGCTATAC | | CCTGGTTCCCTGGATAGTACC |
| BDNF | TCATACTTCGGTTGCATGAAGG | | AGACCTCTCGAACCTGCCC |
| NT3 | AGTTTGCCGGAAGACTCTCTC | | GGGTGCTCTGGTAATTTTCCTTA |
| NT4 | | TGAGCTGGCAGTATGCGAC | CAGCGCGTCTCGAAGAAGT |
| ACSL3 | | AACCACGTATCTTCAACACCATC | AGTCCGGTTTGGAACTGACAG |
| ACSL1 | | GCAACCGGGTCAAGTTGGT | CAAGTCGTTGGAGTAGTTGGG |
| ACSL4 | | ATATTCGTCACCACTCACA | AACCTTGCTCATAACATTCTT |
| GPX4 | | CGATACGCTGAGTGTGGTTT | CGGCGAACTCTTTGATCTCTT |
| GPX4 site1 | | CAAGTGGAACTTCACCAAG | CACACACTTGTGGAGCT |
| GPX4 site2 | | TGCGCGCTCCATGCACGAGTTT | CACGTTGGTGACGATGCACACGAA |
| GPX4 site3 | | CTGCCTGCAAACCTGCTGGT | CTGTTTATTCCCACAAGGTAG |
| FABP5 | | AAAGAGCTAGGAGTAGGACTGG | TGTTGCCATCACACGTAATGA |
| CYPLA2 | | AGTACATCTCCTTAGCCCCAG | GGGTCCGGGTGGATTCTTC |
| CYP2J6 | | TTAGCCACGATCTGGGCAG | CTGGGGGATAGTTCTTGGGG |
| CYP2E1 | | CGTTGCCTTGCTTGTCTGGA | AAGAAAGGAATTGGGAAAGGTCC |
| CYP2C | | ATCTGGTCGTGTTCCTAGCG | CAGTAGGCTTTGAGCCCAAATA |
| ELOVL2 | | CACGTACCTGCTCTCGATATGG | TGTGATTGCGAGGTTATACAAGG |
| ELOVL3 | | CGGATGACGCCGTAGTCAG | GGACGCTTACGCAGGATGAT |
| ELOVL5 | | ATGGAACATTTCGATGCGTCA | GTCCCAGCCATACAATGAGTAAG |
| PLA2G4 | | CAGCACATTATAGTGGAACACCA | AGTGTCCAGCATATCGCCAAA |
| APOE | | CTGACAGGATGCCTAGCCG | CGCAGGTAATCCCAGAAGC |
| COX1 | | ATGAGTCGAAGGAGTCTCTCG | GCACGGATAGTAACAACAGGGA |
| Fads2 | | TCATCGGACACTATTCGGGAG | GGGCCAGCTCACCAATCAG |
| Alox12 | | ACCTCAGACAATAGCAGCGGA | TCAACGTCCATTCAAAGTCCAG |
| Alox15 | | GGCTCCAACAACGAGGTCTAC | CCCAAGGTATTCTGACACATCC |
| LRG1 | | TTGGCAGCATCAAGGAAGC | CAGATGGACAGTGTCGGCA |
| ANXA2 | | ATGTCTACTGTCCACGAAATCCT | TGACTGACCCGTAGGCACTT |
| SREBP1 | | TGACCCGGCTATTCCGTGA | CTGGGCTGAGCAATACAGTTC |

Supplementary Table 3-2 METTL3 SIRNA

| Gene name | sense（5'-3'） | antisense（5'-3'） |
| --- | --- | --- |
| METTL3 | GCCAAGGAACAAUCCAUUGUUTT | AACAAUGGAUUGUUCCUUGGCTT |
| METTL3 | CGUCAGUAUCUUGGGCAAGUUTT | AACUUGCCCAAGAUACUGACGTT |
